# 8-Br-cGMP activates HSPB6 and increases the antineoplastic activity of quinidine in prostate cancer

**DOI:** 10.1038/s41420-024-01853-3

**Published:** 2024-02-19

**Authors:** Yuankang Feng, Zhenlin Huang, Fubo Lu, Liang Song, Ruoyang Liu, Yu Zhang, Ningyang Li, Xu Han, Xiang Li, Keqiang Li, Budeng Huang, Guoqing Xie, Abao Guo, Jinjian Yang, Zhankui Jia

**Affiliations:** Department of Urology, the First Affiliated Hospital of Zhengzhou University, Zhengzhou, 450052 China

**Keywords:** Prostate cancer, Cancer therapy

## Abstract

Heat shock protein family B [small] member 6 (HSPB6), widely found in various muscles, has been recently identified as a tumor suppressor gene. However, its role in prostate cancer remains unexplored. Herein, we investigated the expression of HSPB6 in prostate cancer and its association with prognosis. Our findings revealed that *HSPB6* downregulation in prostate cancer correlated with a poor prognosis. Moreover, we discovered that HSPB6 can be phosphorylated and activated by 8-Br-cGMP, leading to apoptosis in prostate cancer cells by activating Cofilin. Additionally, we demonstrated that knocking down E2F1 by quinidine administration enhances the transcriptional level of HSPB6. Furthermore, we evaluated the combination of quinidine and 8-Br-cGMP as a potential therapeutic strategy for prostate cancer. Our results revealed that the combined treatment was more effective than either treatment alone in inhibiting the growth of prostate cancer through the *HSPB6* pathway, both in vitro and in vivo. Overall, our study provides compelling evidence that HSPB6 suppresses malignant behavior in prostate cancer by inducing apoptosis. The combination of quinidine and 8-Br-cGMP emerges as a promising approach for the treatment of prostate cancer.

## Introduction

Prostate cancer (PC) is a significant global health concern that impacts men’s longevity, with a high long-term survival rate for localized cases [1]. Early detection and standardized therapy offer a favorable prognosis for this patient population. Treatment efficacy for localized prostate cancer is based on early diagnosis and surgery. However, advanced and recurrent prostate cancer relies heavily on medical therapies like androgen deprivation therapy (ADT), chemotherapy, or novel androgen signaling-targeted agents [2].

Apoptosis, a programmed cell death process involving the Caspase family, especially Caspase3 [3], plays a crucial role in various cellular biological processes. Its function has garnered significant attention in relation to several diseases. Over the years, apoptosis has been associated with tumor cell proliferation, metabolism [4, 5], cancer treatment and prevention [6, 7], prognostic assessment of cancer patients, and various other aspects [8]. It is now understood that apoptosis is indispensable in prostate cancer, providing valuable avenues for clinical treatment of intermediate and advanced PC cases [9]. Current evidence suggests that the evasion of apoptosis is a significant factor contributing to drug resistance in prostate cancer cells [10]. Thus, future research on apoptosis holds promise for discovering novel treatments for prostate cancer.

Small heat shock proteins (sHSPs), with relative molecular masses ranging from 12 to 43 kDa, serve as crucial molecular chaperones vital in various life activities. Among these sHSPs, Heat shock protein family B [small] member 6 (HSPB6), also known as HSPB20, exhibits widespread expression in diverse muscle types, including airway, vascular, prostate, bladder, uterine smooth muscle, skeletal muscle, and cardiac muscle [11]. Studies have demonstrated that HSPB6 exhibits tumor-suppressive properties and can inhibit the progression of various cancers, such as liver cancer, ovarian cancer, and so on [12–14]. However, there is a significant gap in our understanding of the role of HSPB6 in prostate cancer.

Previous investigations have revealed hypermethylation of the *HSPB6* promoter affects its function [15], but the transcriptional regulation of *HSPB6* in cancer remains unexplored. To gain insights into the regulatory mechanism of HSPB6 expression, we aimed to identify a novel transcription factor that governs HSPB6 expression. Aberrant activation of E2F Transcription Factor 1 (E2F1) in tumors has been associated with a poor prognosis, which has also been established in prostate cancer [16, 17]. Nonetheless, no studies have investigated its potential relationship with HSPB6.

Herein, we verified that E2F1 negatively regulates the expression of HSPB6. Silencing E2F1 upregulated HSPB6 expression, promoting apoptosis in prostate cancer cells. This effect was further potentiated by cGMP. Additionally, we discovered that quinidine could reduce the expression of E2F1 in prostate cancer, providing theoretical support for the combination of quinidine and 8-Br-cGMP in clinical applications.

## Results

### The expression of *HSPB6* is downregulated in prostate cancer

**Fig. 1:**
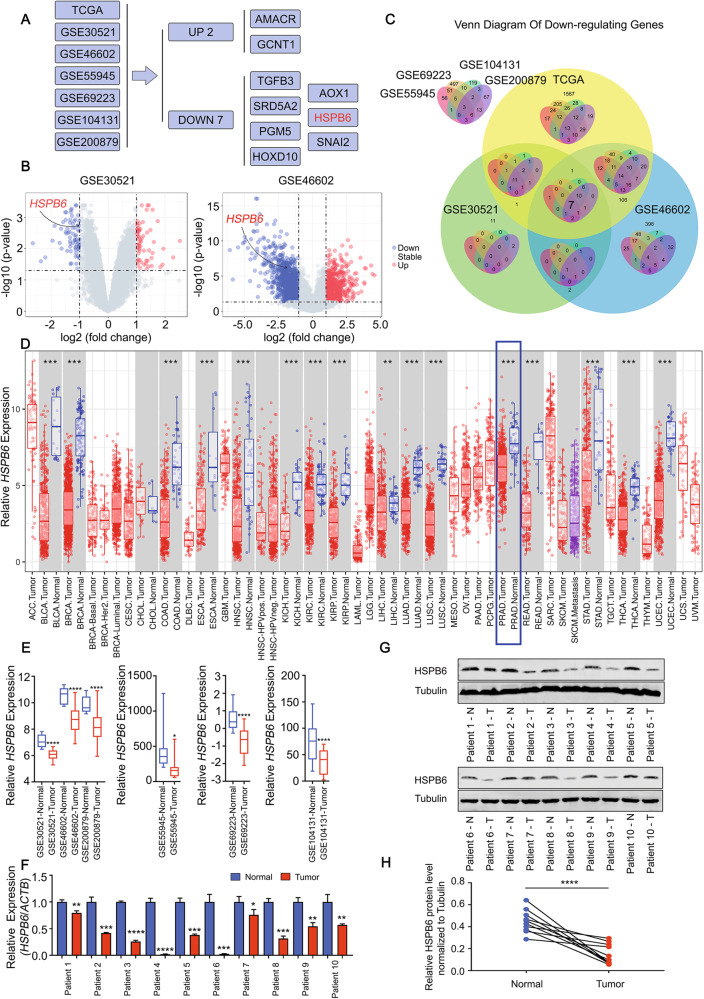
The expression of HSPB6 is downregulated in prostate cancer. **A** The process of screening HSPB6 as a research object. **B**, **C** Differentially expressed genes between prostate cancer and normal tissue from the TCGA database and the GEO database (GSE30521, GSE46602, GSE55945, GSE69223, GSE104131, and GSE200879), in the form of a Volcano diagram (**B**) and a Venn plot (**C**), respectively. **D** Expression of *HSPB6* in different cancers from the TCGA database. *P* < 0.01 as “**“, *P* < 0.001 as “***“, unpaired t-test. **E** Expression of *HSPB6* in prostate cancer from the GEO database (GSE30521, GSE46602, GSE55945, GSE69223, GSE104131, and GSE200879). *P* < 0.05 as “*“, *P* < 0.0001 as “****“, unpaired t-test. **F**–**H** RT-qPCR (**F**) and Western blot analysis (**G**, **H**) showed the HSPB6 expression in prostate cancer and normal tissues. ACTB and GAPDH served as internal references. *P* < 0.0001 as “****“, paired *t* test.

The progression of prostate cancer is a complex biological process involving crucial players such as *p53*, *AR*, and *PTEN*. However, the treatment of prostate cancer, especially in advanced-stage cases, remains challenging. Therefore, it is essential to explore new therapeutic targets. Through the analysis of seven datasets (TCGA, GSE30521, GSE4602, GSE55945, GSE69223 GSE104131, and GSE200879), we identified two upregulated genes (*AMACR* and *GCNT1*) and seven downregulated genes (*TGFB3*, *SRD5A2*, *PGM5*, *HOXD10*, *AOX1*, *HSPB6*, and *SNAI2*) in prostate cancer compared to normal tissue (Fig. 1A–E, Supplementary Fig. 1A, B). Besides *HSPB6*, the above genes have been extensively studied in prostate cancer, with their mechanisms relatively well understood. Due to the lack of comprehensive knowledge of *HSPB6* in prostate cancer, we selected it for a future study. Subsequently, we collected tissue samples from patients and conducted validation in mRNA and protein levels, confirming that HSPB6 expression is lower in prostate cancer than in normal prostate tissue (Fig. 1F–H). The downregulation of HSPB6 expression in prostate cancer suggests it may function as a tumor suppressor gene in this context.

### Prostate cancer patients with lower *HSPB6* expression have a worse prognosis

**Fig. 2:**
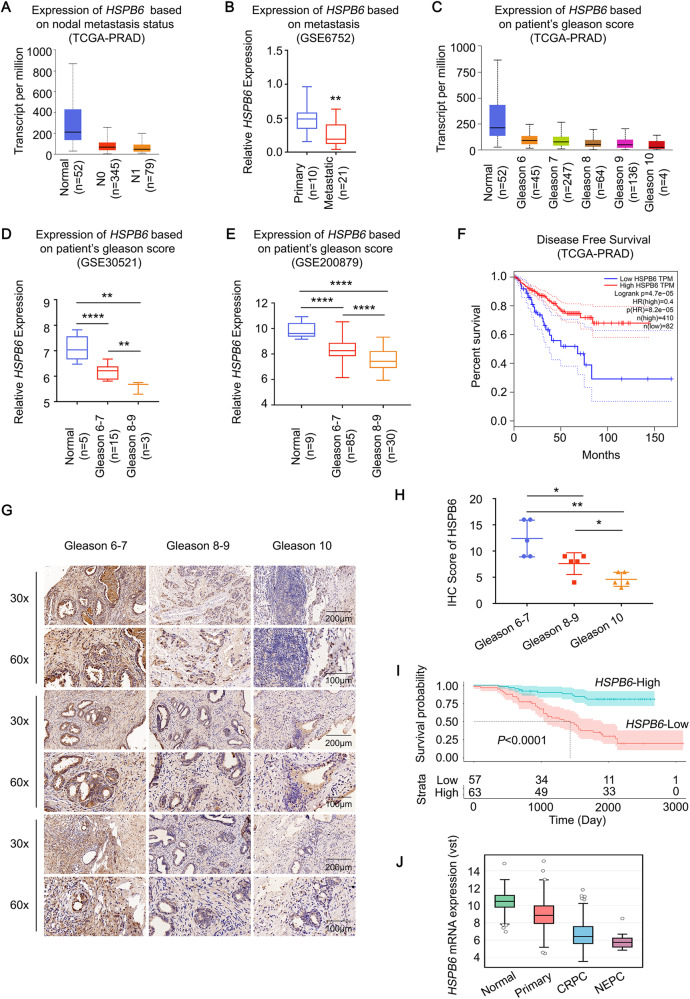
Low expression of *HSPB6* is associated with a poor prognosis for prostate cancer. **A**, **B** Expression of *HSPB6* in metastatic prostate cancer and primary prostate cancer. The data comes from the TCGA database and GEO database (GSE6752). **C**–**E** Expression of *HSPB6* in prostate cancer with different Gleason scores from TCGA database and GEO database (GSE30521 and GSE200879). **F** Disease-free survival analysis for patients with high and low expression of *HSPB6* from TCGA database. **G**, **H** Images (**G**) and dot plots (**H**) of *HSPB6* staining using tissue sections based on Gleason score (prostate cancer: *n* = 15, *P* < 0.05 as “*“, *P* < 0.01 as “**“, unpaired *t* test). **I** Overall survival analysis for patients with high and low expression of *HSPB6* (RT-qPCR) using the cutoff value. **J** Expression of *HSPB6* in CRPC, NEPC, and primary prostate cancer.

In this study, we aimed to investigate the correlation between *HSPB6* expression in prostate cancer and patient prognosis. Our analysis involved examining prostate cancer patients with different grades and stages of the disease. Initially, we observed that the expression of *HSPB6* was significantly lower in metastatic prostate cancer (lymph node metastases or distant metastases) compared to non-metastatic prostate cancer (Fig. 2A, B). Additionally, we divided prostate cancer into groups based on the Gleason score and noticed that the expression of *HSPB6* progressively decreased with higher Gleason scores (Fig. 2C–E, Supplementary Fig. 1C, D). To evaluate disease-free survival (DFS), we utilized data from TCGA database and conducted a DFS analysis, which revealed that prostate cancer patients with lower *HSPB6* expression had poorer disease-free survival rates (Fig. 2F). Moreover, analysis of immunohistochemical data from the HPA database revealed HSPB6 expression in high-grade prostate cancer was lower than in low-grade prostate cancer (Supplementary Fig. 1E, F). To strengthen our analysis, we randomly selected 15 patients’ tissues with different Gleason scores for immunohistochemical analysis and obtained consistent results (Fig. 2G, H). Furthermore, we conducted qRT-PCR on tissue samples from all 120 patients and overall survival analysis based on the optimal cutoff value, which demonstrated that low *HSPB6* expression correlated with a worse prognosis (Fig. 2I, Supplementary Fig. 1G). The clinical information analysis indicated that HSPB6 expression was negatively correlated with Gleason score, T classification, lymph node metastasis, and distant metastasis, highlighting its significance as a clinical indicator (Table 1). Interestingly, we additionally found that *HSPB6* expression in castration-resistant prostate cancer (CRPCs) and neuroendocrine prostate cancer (NEPCs) was lower than in primary prostate cancer (Fig. 2J). The WB results indicate a significant increase in the expression levels of Cleaved-Caspase3 and a noticeable decrease in PCNA (a marker protein of proliferation) expression among patients exhibiting high HSPB6 expression. In contrast, there were no discernible differences in the expression levels of N-cadherin, E-cadherin, and Vimentin (Supplementary Fig. 1H, I). This further substantiates that prostate cancer tissues with high HSPB6 expression demonstrate elevated levels of apoptosis and reduced proliferation, yet the relationship with migration remains unclear.

**Table 1:** Relationship between the expression of *HSPB6* in prostate cancer and clinicopathological parameters.

Characteristics	*N*	*HSPB6*-high	*HSPB6*-low	*χ*^2^	*P*
Age					
≥70	62	33	29	0.027	0.869
<70	58	30	28		
Gleason score					
≥8	43	15	28	8.339	0.004*
<8	77	48	29		
T classification					
T3, T4	73	31	42	7.525	0.006*
T1, T2	47	32	15		
Lymph node metastasis					
Present	41	14	27	8.413	0.004*
Absent	79	49	30		
Distant metastasis					
Present	52	19	33	9.375	0.002*
Absent	68	44	24		

*Means *P* < 0.05

### *HSPB6* inhibits the progression of prostate cancer by promoting apoptosis of prostate cells

**Fig. 3:**
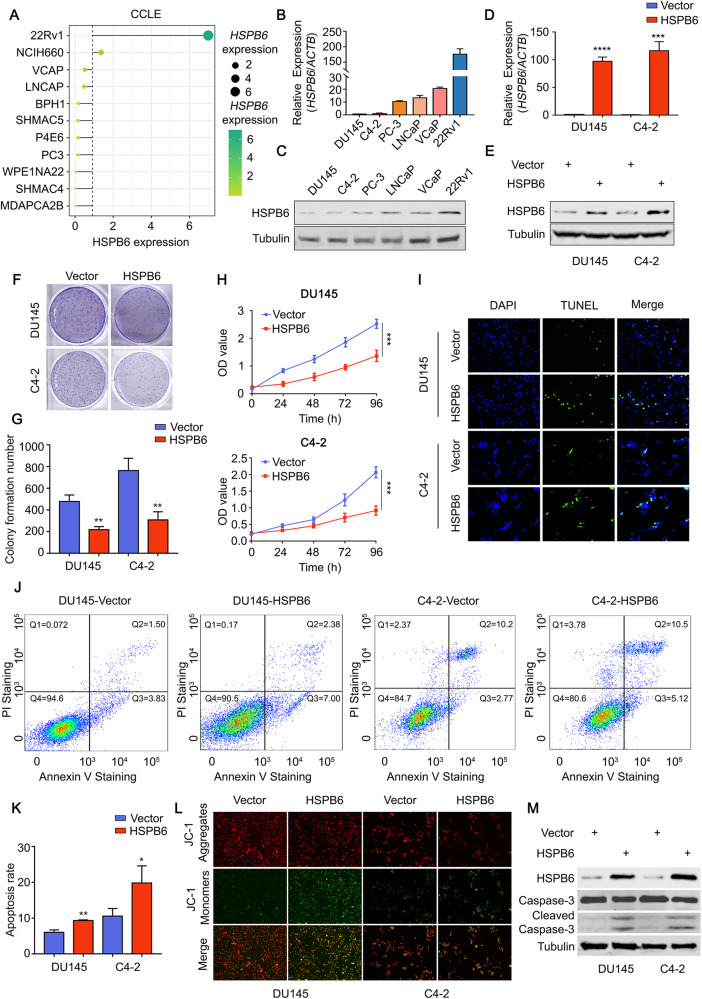
HSPB6 induces apoptosis in prostate cancer. **A** *HSPB6* expression in different prostate cancer cell lines in the CCLE database. **B**, **C** RT-qPCR (**B**) and Western blot analysis (**C**) were used to detect the expression of HSPB6 in different prostate cancer cell lines. **D**, **E** RT-qPCR (**D**) and Western blot analysis (**E**) showed the efficiency of overexpressing *HSPB6* in DU145 and C4-2. (*P* < 0.001 as “***“, *P* < 0.0001 as “****“, unpaired *t* test.) **F**–**H** Colony formation assays (**F**, **G**) and CCK8 (**H**) showed the proliferation ability of DU145 and C4-2 after overexpression of *HSPB6*. (*P* < 0.01 as “**“, *P* < 0.001 as “***“, unpaired *t* test, ANOVA.) **I**–**K** TUNEL (**I**) and flow cytometry (**J**, **K**) showed the apoptosis levels of DU145 and C4-2 after overexpression of *HSPB6*. (*P* < 0.05 as “*“, *P* < 0.01 as “**“, unpaired *t* test.) **L** Mitochondrial membrane potential levels of DU145 and C4-2 after overexpression of *HSPB6* were shown by JC-1 staining. **M** Western blot analysis showed that the expression of the apoptosis marker Cleaved-Caspase-3 after overexpression of *HSPB6*.

In this study, we investigated the role of HSPB6 in prostate cancer progression. We hypothesized that elevated *HSPB6* expression in prostate cancer could inhibit malignant tumor behavior. To test this hypothesis, we constructed an *HSPB6* overexpression plasmid and selected two prostate cancer cell lines, DU145 and C4-2, with the lowest *HSPB6* expression for further investigation (Fig. 3A–C). After confirming the successful transfection of the *HSPB6* plasmids (Fig. 3D, E), we conducted a series of experiments to understand the function of *HSPB6*. Firstly, we observed that the proliferation of prostate cancer cells was significantly inhibited after *HSPB6* overexpression (Fig. 3F–H), supporting the notion that *HSPB6* acts as a tumor suppressor. Subsequent apoptosis-related experiments revealed that *HSPB6* overexpression led to a higher number of broken DNA and an increased proportion of apoptotic cells compared to the control group (Fig. 3I–K, Supplementary Fig. 2A). Additionally, we observed a reduction in mitochondrial membrane potential in the *HSPB6* overexpression group compared to the control group (Fig. 3L, Supplementary Fig. 2B), indicating that HSPB6 induced apoptosis in prostate cancer cells. Furthermore, we found a significant increase in the expression of Cleaved-Caspase3 after *HSPB6* overexpression (Fig. 3M), providing further evidence of apoptosis induction by HSPB6. To strengthen our findings, HSPB6 was knocked down in the 22Rv1 cell line, which yielded similar results, supporting the role of HSPB6 in promoting apoptosis in prostate cancer cells (Supplementary Fig. 2C–N).

### cGMP activates *HSPB6* and promotes apoptosis in prostate cancer cells

**Fig. 4:**
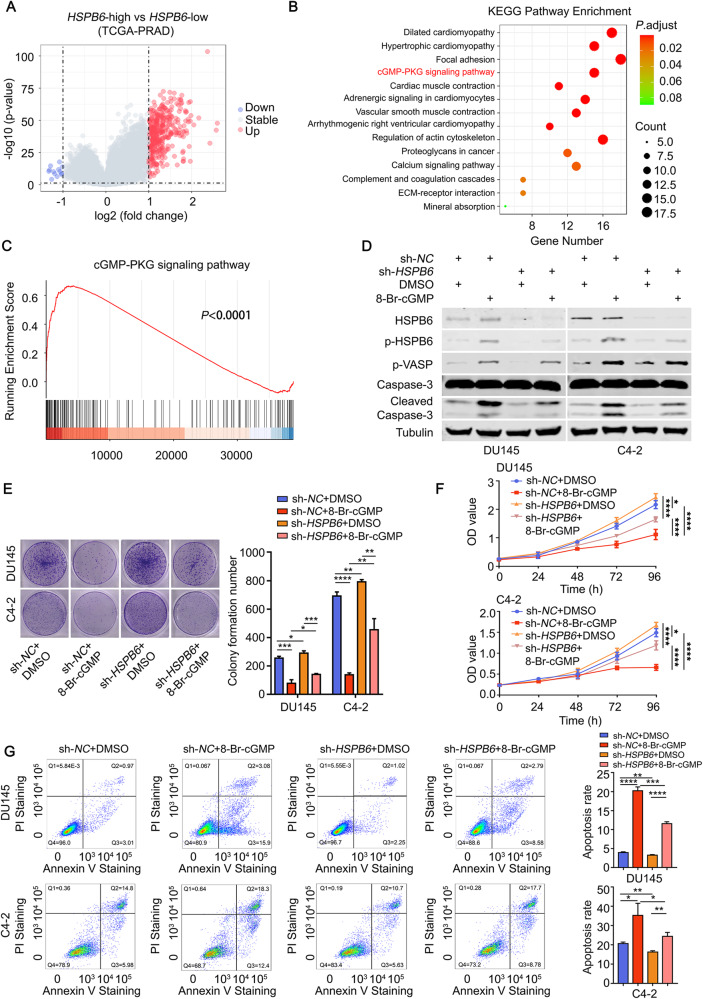
8-Br-cGMP induces apoptosis by phosphorylating HSPB6. **A** Differentially expressed genes between *HSPB6*-High PRAD and *HSPB6*-Low PRAD from TCGA database. **B** The results of the KEGG analysis of the differential genes. **C** The image represented the results of the GSEA analysis of the differential genes. **D** Western blot analysis showed that the expression of p-HSPB6, p-VASP, and Cleaved-Caspase-3 after knockdown *HSPB6* and (or) supplementation with 8-Br-cGMP (1 mM) (p-VASP as positive control). **E**, **F** Colony formation assays (**E**) and CCK8 (**F**) showed the proliferation ability of DU145 and C4-2 after knockdown *HSPB6* and (or) supplementation with 8-Br-cGMP (1 mM). (*P* < 0.05 as “*“, *P* < 0.01 as “**“, *P* < 0.001 as “***“, *P* < 0.0001 as “****“, unpaired t-test, ANOVA.) **G** Flow cytometry showed the apoptosis levels of DU145 and C4-2 after knockdown *HSPB6* and (or) supplementation with 8-Br-cGMP (1 mM). (*P* < 0.05 as “*“, *P* < 0.01 as “**“, *P* < 0.001 as “***“, *P* < 0.0001 as “****“, unpaired *t* test).

To further investigate the specific mechanism underlying the function of *HSPB6*, we conducted a single-gene analysis of *HSPB6*. Using optimal cutoff values, we divided the prostate cancer data from TCGA into high-expression and low-expression groups and performed differential gene analysis (Fig. 4A, Supplementary Fig. 3A). Subsequently, we conducted Kyoto Encyclopedia of Genes and Genomes (KEGG) and Gene Set Enrichment Analysis (GSEA), focusing on the cGMP-PKG signaling pathway after excluding non-cancer-related pathways (Fig. 4B, C). Our analysis revealed a significant association between *HSPB6* and 15 genes in the cGMP-PKG signaling pathway (Supplementary Fig. 3B–D). Guo-Chang Fan et al. previously suggested that cGMP-PKG could phosphorylate HSPB6 in the circulatory system [11], leading us to speculate that a similar role may be played in prostate cancer, supported by the positive correlation between *HSPP6* expression and genes of the cGMP-PKG signaling pathway. To explore the impact of cGMP on apoptosis in prostate cancer cells, we utilized 8-Br-cGMP, a cGMP analog, as our research subject. Our experiments demonstrated that low concentration (200 μM) 8-Br-cGMP had minimal effect on the apoptosis of prostate cancer cells, whereas at high concentration (1 mM), it significantly induced apoptosis (Supplementary Fig. 3E, F). Subsequently, we treated prostate cancer cells with 1 mM 8-Br-cGMP, which resulted in the phosphorylation of HSPB6 and increased expression of Cleaved-Caspase3. These effects were inhibited after knocking down *HSPB6* (Fig. 4D). Additional functional experiments supported that 8-Br-cGMP could phosphorylate HSPB6 and induce apoptosis in prostate cancer cells (Fig. 4E–G). As a supplementary experiment, we supplemented 200 μM 8-Br-cGMP after overexpressing *HSPB6*. The results showed that although 200 μM 8-Br-cGMP did not significantly induce apoptosis in prostate cancer cells alone, it significantly increased the apoptosis of *HSPB6*-induced prostate cancer cells (Supplementary Fig. 3E, F). This further demonstrates that cGMP activates HSPB6 and promotes apoptosis in prostate cancer cells.

### p-HSPB6 replaces p-Cofilin from YWHAG to induce apoptosis

It is well-established that *HSPB6* can induce apoptosis in prostate cancer cells, but the underlying mechanism remains unclear. Using three databases, we analyzed proteins that interacted with HSPB6 and identified two proteins, 14-3-3γ (YWHAG) and BAG3, with YWHAG showing the most pronounced statistical significance (Supplementary Fig. 3A–D). A PubMed search using “HSPB6” and “14-3-3” as keywords revealed a study by Maria V Sudnitsyna et al., which indicated that p-HSPB6 could replace p-Cofilin from 14-3-3ζ [18]. Therefore, we speculated that YWHAG may have a similar function to 14-3-3ζ. Interestingly, we found that YWHAG could interact with both HSPB6 and Cofilin (Supplementary Fig. 3E).

**Fig. 5:**
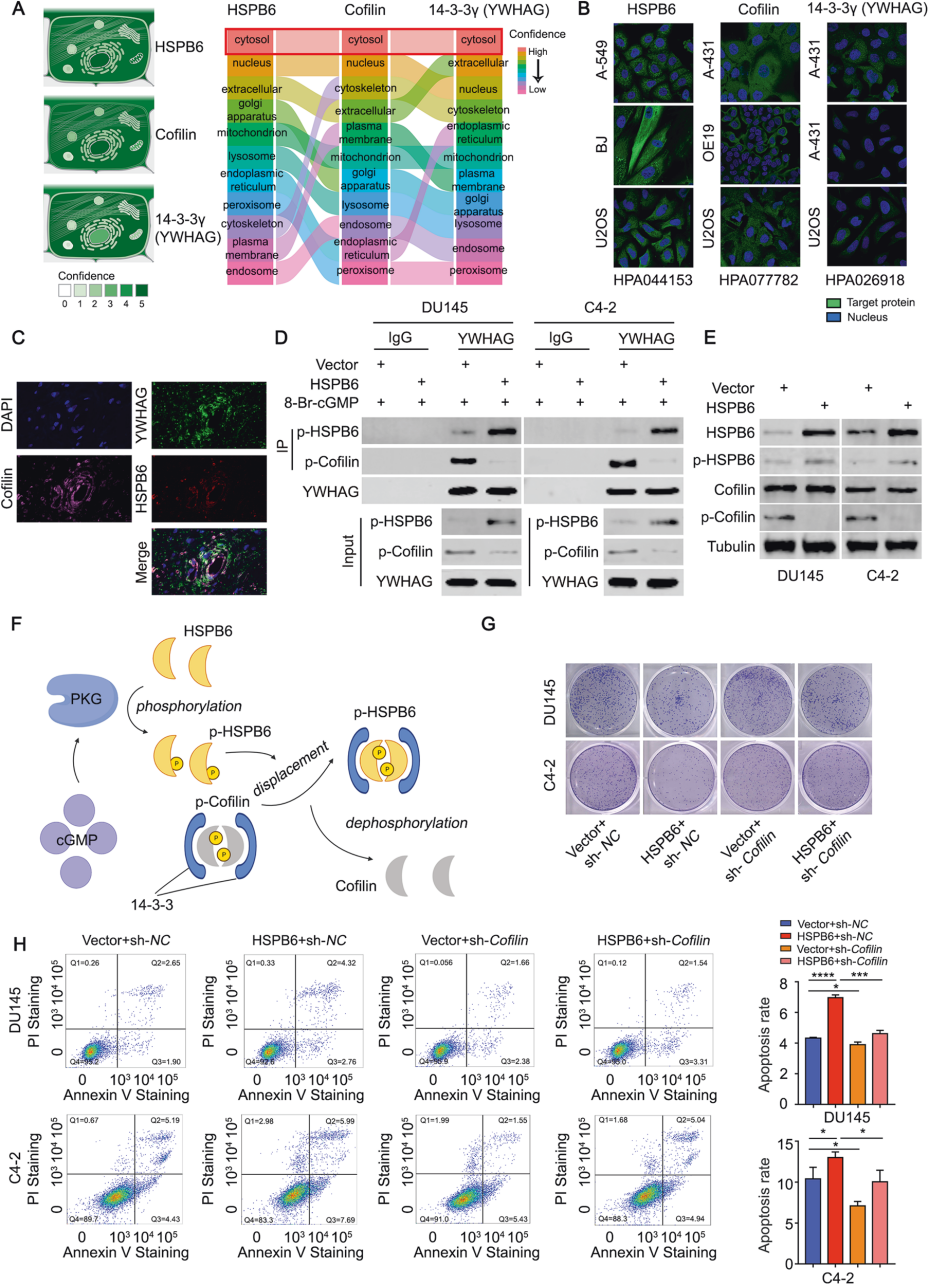
p-HSPB6 replaces p-Cofilin from YWHAG to induce apoptosis. **A** Subcellular localization of HSPB6, Cofilin, and YWHAG from the GeneCards database. **B** IF staining results of HSPB6, Cofilin, and YWHAG in different cells from the HPA database. **C** IF staining results of HSPB6, Cofilin, and YWHAG in prostate cancer. **D** Co-IP results showed the binding of YWHAG to p-HSPB6 and p-Cofilin with or without overexpression of *HSPB6*. **E** Western blot analysis showed that the expression of HSPB6, p-HSPB6, Cofilin, and p-Cofilin changed after overexpression of *HSPB6*. **F** The mechanism by which p-HSPB6 replaces p-Cofilin from YWHAG. **G** Colony formation assays showed the proliferation ability of DU145 and C4-2 after the knockdown of *Cofilin* and (or) overexpression of *HSPB6*. **H** Flow cytometry showed the apoptosis levels of DU145 and C4-2 after the knockdown of *Cofilin* and (or) overexpression of *HSPB6*. (*P* < 0.05 as “*“, *P* < 0.001 as “***“, *P* < 0.0001 as “****“, unpaired t-test).

Moreover, we observed that HSPB6, Cofilin, and YWHAG have similar subcellular localization within cells (Fig. 5A–C). To further investigate this, we performed Co-IP and confirmed that p-HSPB6 can replace p-Cofilin with YWHAG (Fig. 5D). Studies have indicated that the 14-3-3 family can protect the phosphorylation of Cofilin [19], whereas dephosphorylated Cofilin can induce apoptosis of cells [20]. Western blot analysis showed that the total Cofilin expression remained unchanged after HSPB6 overexpression, but the expression of phosphorylated Cofilin decreased (Fig. 5E). We have presented a schematic diagram of the specific mechanism in Fig. 5f. The results of the colony formation assay, CCK8 assay, and flow cytometry also demonstrated that *HSPB6*-induced apoptosis was significantly inhibited after knocking down Cofilin (Fig. 5G, H, Supplementary Fig. 4F, G). In conclusion, these findings suggest that p-HSPB6 replaces p-Cofilin with YWHAG to induce apoptosis.

### E2F1 promotes the transcription of *PROSER3* while inhibiting the transcription of *HSPB6*

**Fig. 6:**
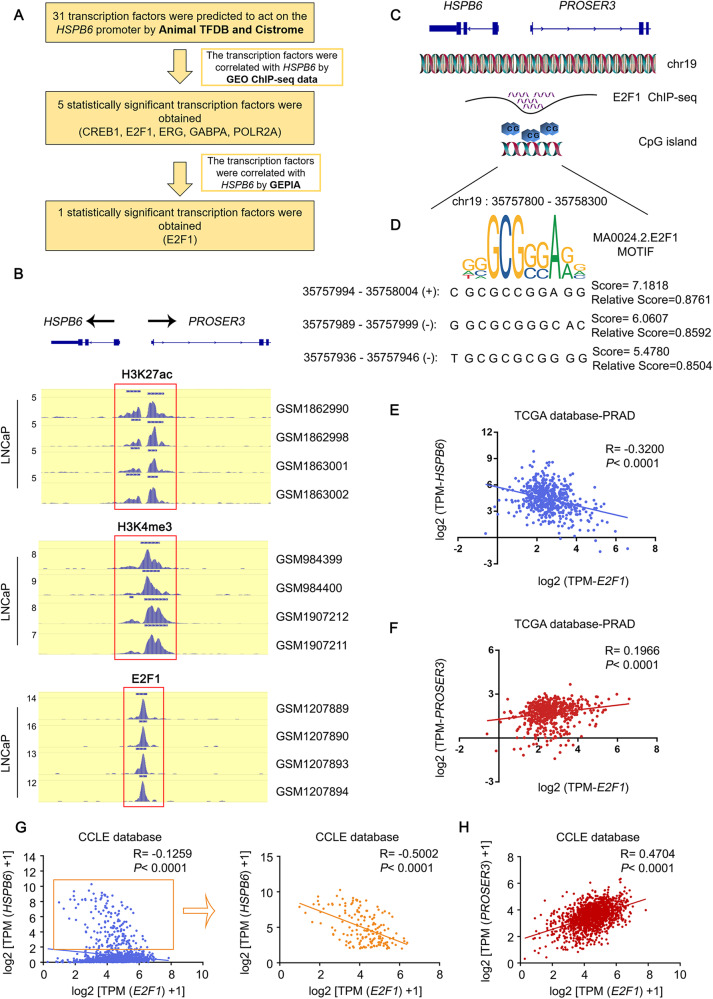
E2F1, as a transcription factor, predictably regulates the expression of *HSPB6* and *PROSER3*. **A** The process of screening transcription factor E2F1. **B** The ChIP-seq results showed the binding of E2F1 to the *HSPB6* promoter region. **C** Schematic diagram of the HSPB6 promoter region. **D** The binding site of E2F1 in the *HSPB6* promoter region. **E**, **F** Correlation analysis of *E2F1* with *HSPB6* (**E**) and *PROSER3* (**F**) from the TCGA database. **G**, **H** Correlation analysis of *E2F1* with *HSPB6* (**G**) and *PROSER3* (**H**) from the CCLE database.

To understand the mechanism regulating *HSPB6* expression in prostate cancer, we performed a series of bioinformatics analyses and identified E2F1 as the only factor with a clear correlation with *HSPB6* (Fig. 6A, Supplementary Fig. 5A–F). ChIP-seq results from the GEO database indicated that E2F1 directly binds to the promoter region of *HSPB6* (Fig. 6B). Interestingly, we found that *HSPB6* shares the same promoter region as *PROSER3*. Our analysis predicted three binding sites of E2F1 in the *HSPB6* promoter region from the JASPAR database (Fig. 6C, D). Furthermore, based on data from TCGA and CCLE databases, we observed a significant negative correlation between *E2F1* and *HSPB6* and a positive correlation between *E2F1* and *PROSER3* (Fig. 6E–H). As a result, we hypothesized that E2F1 promotes the transcription of *PROSER3* while inhibiting the transcription of *HSPB6*.

**Fig. 7:**
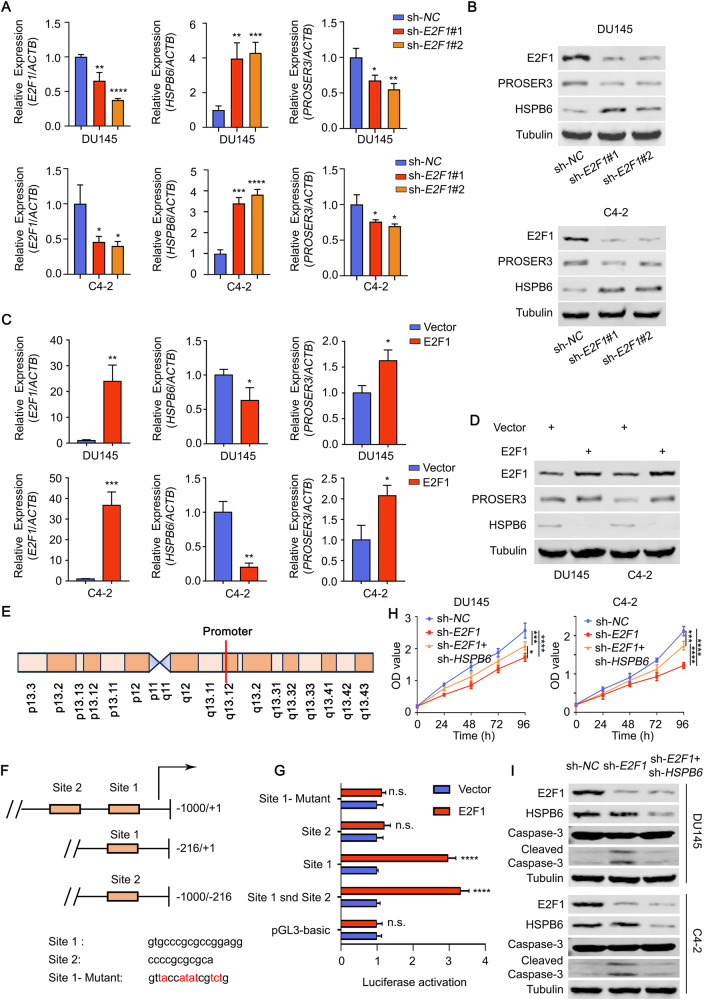
E2F1 regulates the transcription of *HSPB6* and *PROSER3*. **A**, **B** RT-qPCR (**A**) and Western blot analysis (**B**) showed the changes in *HSPB6* and *PROSER3* expression after knockdown *E2F1*. **C**, **D** RT-qPCR (**C**) and Western blot analysis (**D**) showed the changes in *HSPB6* and *PROSER3* expression after overexpression of *E2F1*. **E** Location of *HSPB6* and *PROSER3* promoter on the chromosome. **F** Fluorescent plasmids constructed by different truncations of the *HSPB6* and *PROSER3* promoter. **G** The image represented the fluorescence intensity after co-transfection of different fluorescent plasmids and *E2F1* plasmids. **H** CCK8 showed the proliferation ability of DU145 and C4-2 after overexpression of *E2F1* and *HSPB6*. (*P* < 0.05 as “*”, *P* < 0.001 as “***”, *P* < 0.0001 as “****”, ANOVA.) **I** Western blot analysis showed that the expression of the apoptosis marker Cleaved-Caspase-3 changed after overexpression of *E2F1* and *HSPB6*.

Next, our investigation involved knocking in *E2F1* in mice, leading to a significant and time-dependent decrease in *HSPB6* expression (Supplementary Fig. 5G). Similarly, when E2F1 was knocked down in vitro, *HSPB6* expression increased, while *PROSER3* expression decreased (Fig. 7A, B). As expected, *E2F1* overexpression showed the opposite trend (Fig. 7C, D). Subsequently, we constructed different fluorescent plasmids containing various binding sites of *E2F1* and co-transfected them into 293 T cells along with the *E2F1* plasmid. Our results revealed that Site 1 was the in vivo binding site of E2F1 (Fig. 7E–G).

Finally, we overexpressed *E2F1* and observed its promotion of prostate cancer cell proliferation. In contrast, *PROSER3* did not affect the proliferation of prostate cancer cells (Supplementary Fig. 5H–M). Interestingly, we found that overexpression of *HSPB6* based on *E2F1* overexpression could inhibit the cancer-promoting ability of *E2F1* (Fig. 7H). Previous studies have highlighted that E2F1 accelerates the cell cycle and inhibits apoptosis. However, our study showed that overexpression of *HSPB6* could counter the inhibition of apoptosis induced by E2F1 without affecting E2F1-induced changes in the cell cycle (Fig. 7I, Supplementary Fig. 5N, O).

### The combination of quinidine and 8-Br-cGMP can synergistically inhibit the growth of prostate cancer through *HSPB6*

**Fig. 8:**
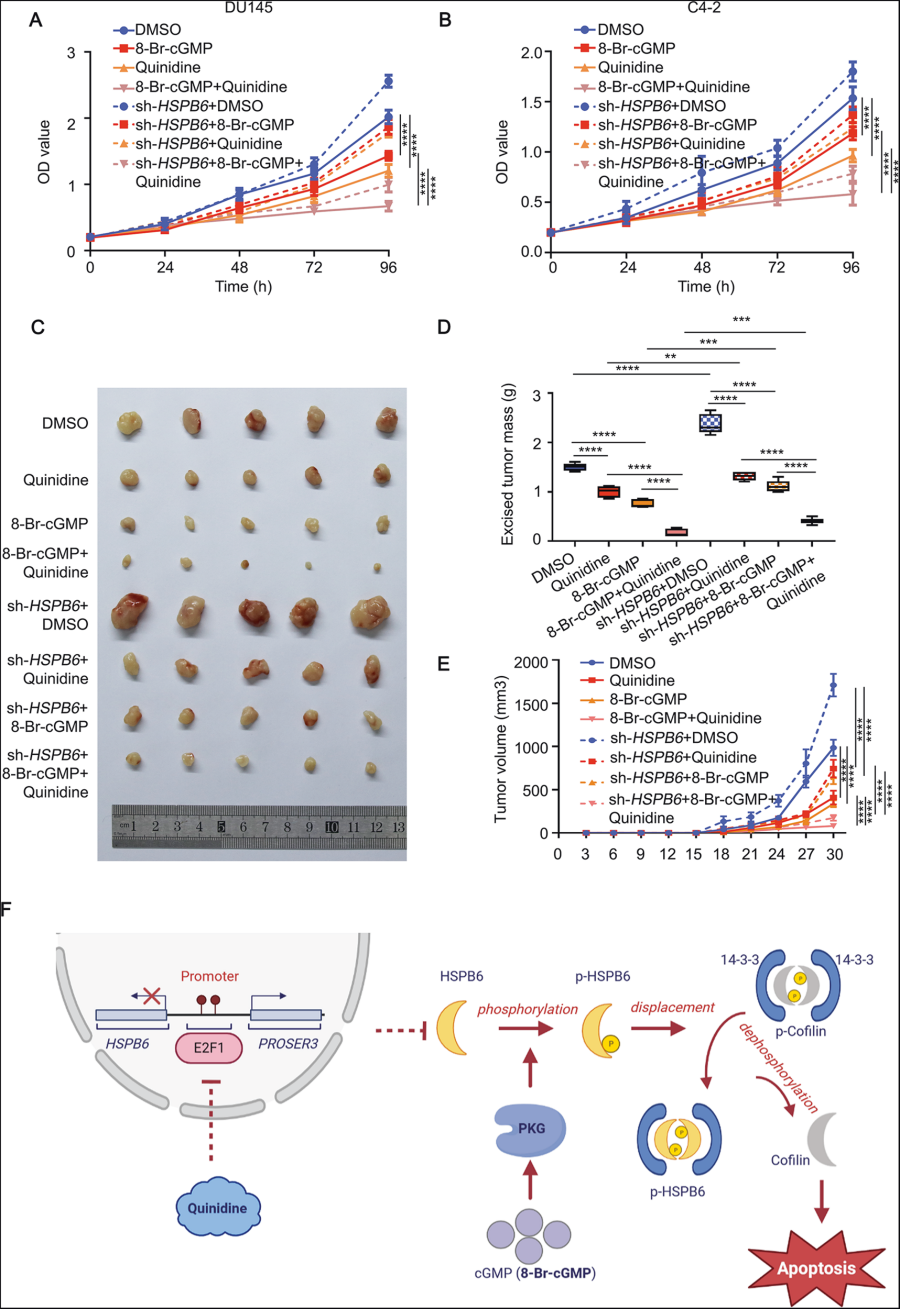
E2F1 regulates the transcription of *HSPB6* and *PROSER3*. **A**, **B** CCK8 showed the proliferation ability of DU145 and C4-2 after supplementation with 8-Br-cGMP and (or) quinidine and (or) knockdown *HSPB6*. (*P* < 0.0001 as “****”, ANOVA). **C**–**E** The image representative of in vivo tumor xenograft model (C4-2) (**C**) performed on SCID mice (*n* = 5) and the volume (**E**) and weight (**D**) statistic of the tumor with 8-Br-cGMP and (or) quinidine and (or) knockdown *HSPB6*. (*P* < 0.01 as “**“, *P* < 0.001 as “***“, *P* < 0.0001 as “****“, unpaired *t* test, ANOVA.) **F** Specific mechanistic diagram of the E2F1/*HSPB6*/Cofilin axis.

Using the DGIdb database and western blot, we found that quinidine significantly inhibited the expression of *E2F1* (Supplementary Fig. 6A–D). We observed an elevation in the protein expression of HSPB6 and Cleaved-Caspase3 upon quinidine addition. Exhilaratingly, upon the addition of quinidine and subsequent HSPB6 knockdown, the expression of Cleaved-Caspase3 was inhibited, resembling the effects observed during *E2F1* and *HSPB6* knockdown. These findings suggest that quinidine’s facilitation of apoptosis may be attributed to its ability to elevate HSPB6 (Supplementary Fig. 6E, F). Additionally, we identified five downstream genes (*IGF1R*, *BMI1*, *RHAMM*, *NuSAP1,* and *PBK*) of E2F1 in prostate cancer [17], and these genes are associated with the survival and proliferation of prostate cancer. Upon the addition of quinidine to prostate cancer cell lines (DU145 and C4-2), we examined the mRNA expression levels of these five genes and *HSPB6*, revealing that *HSPB6*, *RHAMM*, *PBK,* and *NUSAP1* displayed a consistent and statistically significant trend across both cell lines. Surprisingly, in C4-2, we observed a marked decline in genes *RHAMM*, *NuSAP1*, and *PBK* following the addition of quinidine (Supplementary Fig. 6G, H). CCK8 results demonstrated that the combination of quinidine and 8-Br-cGMP in vitro more effectively inhibited the growth of prostate cancer cells, and knocking down *HSPB6* could reverse this effect (Fig. 8A, B). To strengthen our findings, we conducted cell-derived xenograft experiments in SCID mice, which yielded consistent results in vivo (Fig. 7C–F, Supplementary Figs. 7, 8).

Overall, our study revealed that *HSPB6* induces apoptosis by dephosphorylating Cofilin, and its regulation is negatively influenced by *E2F1*. Furthermore, *HSPB6* plays a crucial role in the synergistic cancer suppression of quinidine and 8-Br-cGMP.

## Discussion

In 2023, it is estimated that there will be ~288,300 new cases of prostate cancer in the United States, resulting in ~34,700 deaths and a mortality rate of ~12.04%. Prostate cancer has now become the leading and the second most fatal tumor among men in the United States [21]. Conversely, the incidence of prostate cancer in China is considerably lower than in Europe and the United States. However, due to the large population in China, it is still crucial to pay significant attention to the potential harm caused by prostate cancer [22]. In recent years, there have been numerous reports of prostate cancer developing resistance to new endocrine drugs [23–25] (such as abiraterone, enzalutamide, apalutamide, and so on). Consequently, the clinical treatment of advanced and metastatic prostate cancer has become increasingly challenging. Therefore, there is an urgent need to explore novel treatment approaches for prostate cancer to offer patients more therapeutic options rather than being limited to just a few of the aforementioned drugs. In light of this, our research suggests a promising potential role for the combination of quinidine and 8-Br-cGMP in the treatment of prostate cancer.

*HSPB6*, a crucial member of the heat shock protein family B, plays a significant role in smooth muscle contraction [26]. Similar to other heat shock proteins, HSPB6 exhibits chaperone-like activity and has been shown to prevent heart failure [27, 28]. While research [14, 29] has substantiated its protective effects in certain digestive tract tumors, its role in prostate cancer remains unexplored. Our study found that *HSPB6* expression is downregulated in prostate cancer, and this downregulation inhibits tumor progression by inducing apoptosis. Interestingly, this tumor-suppressing effect can be enhanced by supplementing with 8-Br-cGMP, which increases HSPB6 phosphorylation.

Cofilin is an intracellular actin regulatory protein that exhibits an active state when dephosphorylated, unlike most proteins [30]. The protein 14-3-3 has been observed to protect p-Cofilin from dephosphorylation, thus inhibiting its ability to induce apoptosis [19]. Our study corroborated that p-HSPB6 could replace p-Cofilin in its complex with 14-3-3γ (YWHAG), leading to its activation. This *HSPB6*-induced dephosphorylation of p-Cofilin ensured the functional activation of *HSPB6* and revealed the specific mechanism by which *HSPB6* induces apoptosis.

Furthermore, our findings suggest that E2F1 specifically binds to the promoter region of *HSPB6*, inhibiting its transcription. Notably, the compound quinidine can target E2F1 and decrease its expression. In our experiments, quinidine displayed an inhibitory effect on the growth of prostate cancer cells. Previous research by G C Wishart et al. demonstrated quinidine’s role as a drug-resistance modulator in breast cancer [31]. Tamara Utermark et al. found quinidine could inhibit malignant mesothelioma [32], and Qin Ru et al. reported its capacity to induce apoptosis in human gliomas [33]. Although these studies highlight important discoveries, there is currently no evidence supporting the use of quinidine in the treatment of prostate cancer. Nonetheless, our research provides a theoretical foundation for considering quinidine’s potential in clinical prostate cancer treatment, especially in combination with 8-Br-cGMP.

Our findings offer a novel therapeutic approach for the clinical treatment of prostate cancer, suggesting that future treatment iterations may demonstrate even greater efficacy. Nonetheless, translating medical research into clinical practice presents significant challenges. In the future, we aim to conduct a more comprehensive and systematic evaluation of quinidine’s safety and efficacy in vivo to facilitate its improved application in clinical settings.

## Conclusions

Our research proposes an innovative approach for prostate cancer treatment, focusing on *HSPB6* as a target gene. HSPB6 activates Cofilin and can induce apoptosis in prostate cancer cells. Furthermore, phosphorylating HSPB6 with 8-Br-cGMP enhances its pro-apoptotic capabilities. Knocking down E2F1 at the transcriptional level significantly increases *HSPB6* transcription. Additionally, quinidine reduces the expression of E2F1. Combining quinidine with 8-Br-cGMP leads to a more potent anti-cancer effect. *HSPB6* plays a crucial role in the synergistic cancer suppression of quinidine and 8-Br-cGMP.

## Materials and methods

### Cell lines, cell culture, and transfection

The LNCaP, VCaP, C4-2, DU145, PC-3, 22Rv1, and 293 T cell lines were purchased from the Cell Bank of the Chinese Academy of Sciences (Shanghai, China) (Supplementary Table S1). LNCaP, C4-2, and 22Rv1 cells were cultured in RPMI-1640 medium supplemented with 10% FBS. 293 T and VCaP cells were cultured in DMEM medium supplemented with 10% FBS. DU145 cells were cultured in MEM medium supplemented with 10% FBS. PC-3 cells were cultured in Ham’s F-12 medium supplemented with 10% FBS. The cells were cultured for use in the logarithmic growth phase. JetPRIME^®^ in vitro DNA and siRNA transfection reagent

(Polyplus-transfection, Strasbourg, France) was used for cell transfection, following the manufacturer’s protocol (shRNA sequence information in Supplementary Table S2).

### Data mining

We speculated on the differential expression of HSPB6 and its relationship with E2F1 using six Gene Expression Omnibus (GEO) datasets [34] (GSE6752, GSE30521, GSE46602, GSE55945, GSE69223, GSE104131, and GSE200879; https://www.ncbi.nlm.nih.gov/geo), Cancer Cell Line Encyclopedia (CCLE; https://sites.broadinstitute.org/ccle/) dataset [35] and The Cancer Genome Atlas Program (TCGA; https://portal.gdc.cancer.gov/) dataset [36]. Based on these datasets, we analyzed differentially expressed genes of prostate cancer tissue and normal prostate tissue using the limma R package. As a supplement, The University of Alabama at Birmingham CANcer data analysis Portal [37] (UALCAN; https://ualcan.path.uab.edu/index.html), PCaProfiler [38] (https://www.pcaprofiler.com/) and Tumor IMmune Estimation Resource [39] (TIMER; https://cistrome.shinyapps.io/timer/) databases were also used to analyze prostate cancer data. False positive data were amended by adjusting the *P* value. Hence, the criteria for selecting significant data were set as adjusted *P* < 0.05 along with log fold change (log FC) values greater than 1 or less than −1. Venn plots and volcano plots were used to identify the differentially expressed genes across these GEO datasets. To optimize the relationship between the expression of HSPB6 and the prognostic value of prostate cancer, we supplemented the data with a TCGA cohort, which was analyzed using the Gene Expression Profiling Interactive Analysis (GEPIA) database [40] (http://gepia.cancer-pku.cn/). Finally, the PPI protein network was constructed using the STRING [41] (https://string-db.org/), GENEMANIA [42] (https://genemania.org/), and IntAct [43] (https://www.ebi.ac.uk/intact/home) databases.

### Tissue specimens

One hundred and twenty tissue samples were collected from prostate cancer patients between 2011 and 2020 at the First Affiliated Hospital of Zhengzhou University (Zhengzhou, China). The patients in this study received a definite diagnosis by histopathological examination and had not undergone medical treatment. Written informed consent was obtained from every patient included in the study. The research was approved by the Research Ethics Committee of the First Affiliated Hospital of Zhengzhou University (2023-KY-0636-001).

### Quantitative real-time polymerase chain reaction

Total RNA was extracted using the Total RNA Extraction Kit (Boxbio, Beijing, China). Subsequently, cDNA was synthesized using the NovoScript® Plus All-in-one 1st Strand cDNA Synthesis SuperMix (Novoprotein, Shanghai, China). The gene transcripts of interest were quantitated using the QuantStudio Three Real-Time PCR System (Thermo Fisher) using the NovoStart® SYBR qPCR SuperMix Plus (Novoprotein), with ACTB as an internal control. The primer sequence used during our quantitative real-time polymerase chain reaction (qRT-PCR) test is shown in Supplementary Table 3.

### Immunohistochemistry (IHC)

Patient tissues and xenografts were fixed overnight in 4% paraformaldehyde. Subsequently, the fixed tissue blocks were dehydrated and embedded to a suitable size for slicing. The embedded paraffin blocks were then sliced and placed on a slide. Each slice was labeled with a unique identifier and placed on a baking machine for 30 minutes to adhere firmly to the tissue. Sections were sequentially dewaxed, and antigen retrieval was performed. To prepare the tissue for immunohistochemistry analysis, a PAP pen was used to circle the tissue on the slide, and then a blocking solution was applied. Next, the primary antibody was added and left to incubate overnight at 4 °C. Following the primary antibody incubation, the slides were treated with a secondary antibody. For visualization, a DAB chromogenic solution was added to induce color development. After this staining process, photographs of the slides were taken, and statistical analysis was conducted to assess the results.

### Western blot

The total protein content in tissues and cells was extracted using RIPA buffer (Beyotime, Shanghai, China) supplemented with 1% PMSF (Solarbio) and 1% Phosphatase inhibitor cocktail A (Beyotime). A pre-assessment of protein concentration was then performed using a BCA kit (Solarbio, Beijing, China). We electrophoresis the protein using PAGE gel (Epizyme, shanghai, China) and transfer it to the PVDF membrane. The PVDF membrane containing the protein was closed with a QuickBlock™ Blocking Buffer (Beyotime) for half an hour, then the primary antibody was added and incubated overnight at 4 °C. After washing off the residual primary antibody, the PVDF membrane was incubated with the secondary antibody for one hour in the dark. The Odyssey CLx Infrared Imaging System (Gene Company Limited, Hong Kong, China) was used to detect the target protein bands. The corresponding antibody information is provided in Supplementary Table S1.

### Cell counting kit-8 assay (CCK8)

Approximately 4000 prostate cancer cells were uniformly dispersed in 96-well plates and pre-cultured in an incubator (37 °C, 5% CO_2_) until the cell state was stable. Subsequently, 10 µl of CCK8 solution (Dojindo, Tokyo, Japan) was added to each well. After a 4-hour incubation, the absorbance of each well was measured using a microplate reader (Perlong, Beijing, China) at 450 nm for statistical analysis.

### Colony formation assay

Prostate cancer cells (1000–2000 cells) were evenly distributed in 6-well plates and cultured in a cell culture incubator at 37 °C with 5% CO_2_ for 1–2 weeks. The culture was stopped when single cells grew into 50–200 cell clumps, and the cells were fixed with 4% paraformaldehyde for 1 hour. Following fixation, the cells were washed to remove residual paraformaldehyde and stained using a crystal violet staining solution (Beyotime). The number of cell clones per well was observed and analyzed.

### Annexin V/PI double dyeing flow cytometry

In this study, we used the BD Pharmingen™ FITC Annexin V Apoptosis Detection Kit I (BD Biosciences, Franklin Lakes, NJ, USA) to detect cell apoptosis. Prostate cancer cells were cultured in 6-well plates and subjected to various treatments. Subsequently, the prostate cancer cells were digested and counted using pancreatic enzymes. The cells were then diluted to a concentration of 1 × 10^6^ cells/ml using PBS, and 100 µl of this suspension was transferred to a 5 ml culture tube. Next, 5 µl of FITC Annexin V and 5 µl of PI were added to the culture tube. The cells were gently mixed and incubated for 15 minutes at room temperature (25 °C) while protected from light. Following the incubation, 400 µl of 1× Binding Buffer was added to each culture tube, and the samples were analyzed by flow cytometry within 1 hour.

### TUNEL assay

The TUNEL assay was conducted using the One Step TUNEL Apoptosis Assay Kit (Beyotime). The cells were initially washed with PBS to remove residual medium on the cell surface, followed by fixation with 4% paraformaldehyde for 30 minutes. After washing off the residual paraformaldehyde, the cells were incubated with 0.3% Triton X-100 for 5 minutes. Next, we prepared the chromogenic solution according to the kit’s instructions and added 50 µl per well. The cells were then incubated at 37 °C in a light-protected environment for 60 minutes. After removing the stain, we captured images using a fluorescence microscope and analyzed the results statistically.

### Mitochondrial membrane potential assay

We use the Mitochondrial membrane potential assay kit with JC-1 (Beyotime) to assess the mitochondrial membrane potential. An appropriate amount of JC-1 (200×) was taken and diluted with ultrapure water at a ratio of 8 ml per 50 μl of JC-1 (200×). After thoroughly mixing JC-1, 2 ml of JC-1 staining buffer (5×) was added and mixed to create the JC-1 staining solution. Subsequently, 1 ml of the working solution was added to each well of a sixwell plate and incubated for 20 minutes in darkness. Observations and photography were performed using a fluorescence microscope after the incubation period.

### In vivo tumor xenograft model

Male severe combined immunodeficiency (SCID) mice aged 4–5 weeks were procured from Sibeifu Company (Beijing, China). To meet statistical requirements, 80 mice were randomly divided into eight groups (5 mice per group) to minimize experimental error. C4-2 cells (Transfection of Vector or HSPB6) were diluted to 1 × 10^7^ cells with 50 μL PBS and 50 μL Matrigel (Corning), then subcutaneously injected into the mice. After one week, the mice were intraperitoneally injected with 8-Br-cGMP [44] (50 mg/kg per mouse, every 2 days until day 20) and quinidine [45] (25 mg/kg per mouse, every two days until day 20) individually or in combination. Tumor size was observed and measured every three days, and on day 30, tumors were removed and weighed.

The experimental procedures were carried out following the guidelines established by the National Institutes of Health of China and were approved by the Ethics Committee for Animal Experiments of the First Affiliated Hospital of Zhengzhou University, Zhengzhou, China (2023-KY-0636-001).

### Statistical analysis

All data were analyzed using the following software: GraphPad Prism 8.0 (GraphPad, San Diego, CA, USA), R-4.3.1 (R Core Team, https://www.r-project.org/), and SPSS v. 25.0 (IBM, Chicago, IL, USA). We employed a *t* test or ANOVA to assess statistical significance unless stated otherwise. Each experiment was conducted with three replicates. A *P* value < 0.05 was statistically significant.

### Supplementary information

aj-checklist

Supplementary material

Full and uncropped western blots

## Data Availability

All data generated during this study leading to the findings presented here are included in this published article and its Supplementary Data files. All data are available from the corresponding author upon reasonable request.
