# Supplementary material for: 8-Br-cGMP activates HSPB6 and increases the antineoplastic activity of quinidine in prostate cancer
Source: Cell Death Discov. 2024 Feb 19;10:90. doi: 10.1038/s41420-024-01853-3 (PMC10876707; doi:10.1038/s41420-024-01853-3)
Supplement: Supplementary file 2 — Supplementary material [file 41420_2024_1853_MOESM2_ESM.docx]

**Supplementary Information**

**8-Br-cGMP activates HSPB6 and increases the antineoplastic activity of quinidine in prostate cancer**

*Yuankang Feng, Zhenlin Huang, Fubo Lu, Liang Song, Ruoyang Liu, Yu Zhang, Ningyang Li,* *Xu Han, Xiang Li, Keqiang Li, Budeng Huang, Guoqing Xie, Abao Guo, Jinjian Yang, Zhankui Jia*

**Files included in supplementary information**

**Supplementary Figure 1 （Supplemental to Figure 1, 2）**

**Supplementary Figure 2 （Supplemental to Figure 3）**

**Supplementary Figure 3 （Supplemental to Figure 4）**

**Supplementary Figure 4 （Supplemental to Figure 5）**

**Supplementary Figure 5 （Supplemental to Figure 6, 7）**

**Supplementary Figure 6 （Supplemental to Figure 8）**

**Supplementary Figure 7 （Supplemental to Figure 8）**

**Supplementary Figure 8 （Supplemental to Figure 8）**

**Supplementary Table S1**. Cell lines, antibodies and other reagents and resources

**Supplementary Table S2**. Sequence information of shRNAs

**Supplementary Table S3**. Sequence information of primers for RT-qPCR

**
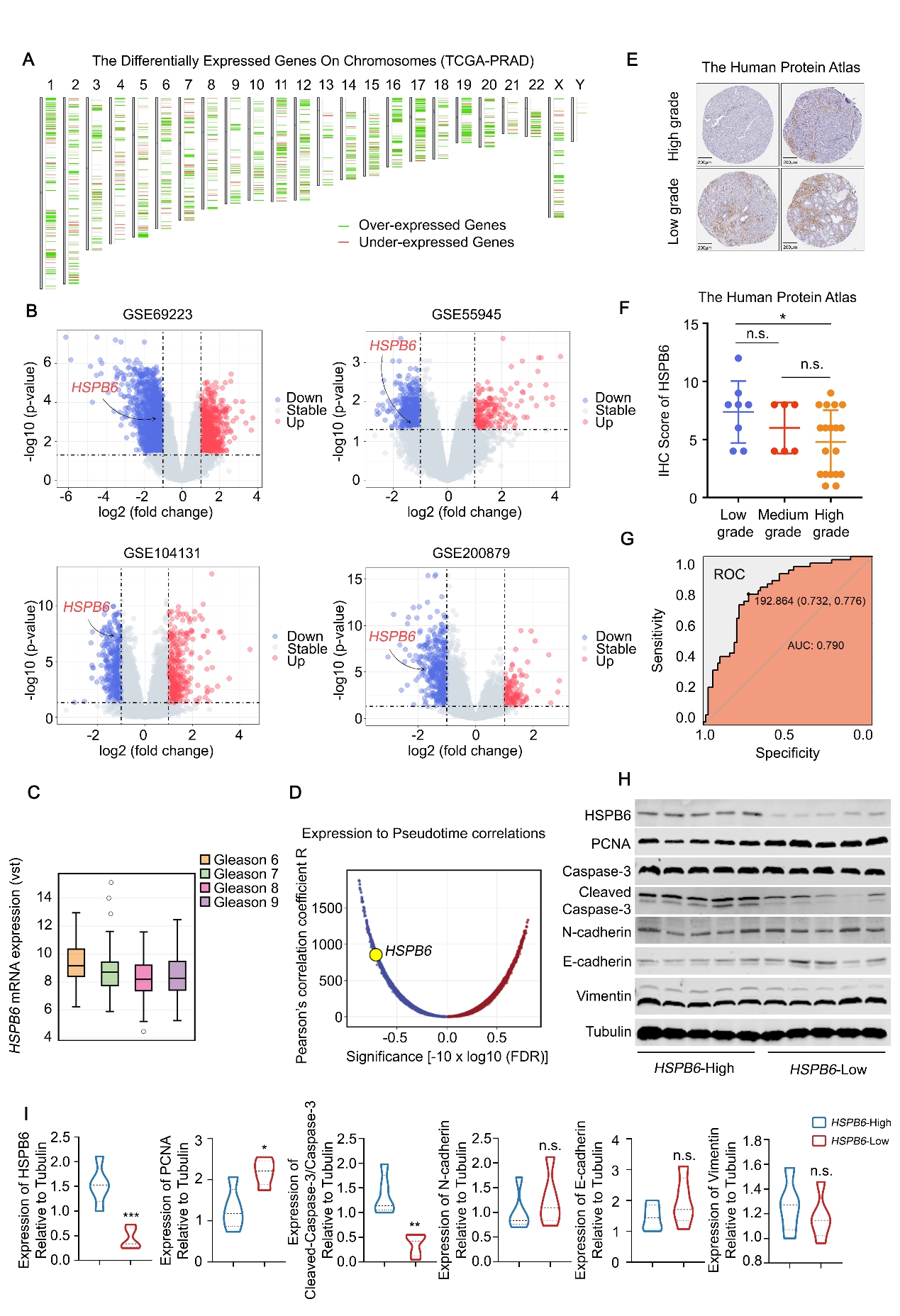
**

**Supplementary Figure 1, Supplemental to Figure 1, 2**

**A** The differentially expressed genes on chromosomes from TCGA-PRAD. **B** The Volcano diagram represented differentially expressed genes between prostate cancer and normal tissue from the GEO database (GSE55945, GSE69223, GSE104131, and GSE200879). **C** Expression of *HSPB6* in prostate cancer with different Gleason scores from PCaProfiler database. **D** *HSPB6* expression to pseudotime correlation. **E, F** IHC staining showed HSPB6 expression in different grades of prostate cancer. (*P* < 0.05 as "*", n.s. means nonspecific, unpaired t-test.) **G** The cut-off value was calculated using the ROC curve of *HSPB6* expression. **H, I** Western blot analysis showed that the expression of the HSPB6, PCNA, Caspase-3, Cleaved Caspase-3, N-cadherin, E-cadherin and Vimentin in *HSPB6*-High prostate cancer and *HSPB6*-Low prostate cancer. (*P* < 0.05 as "*", *P* < 0.01 as "**", *P* < 0.001 as "***", n.s. means nonspecific, unpaired t-test.)


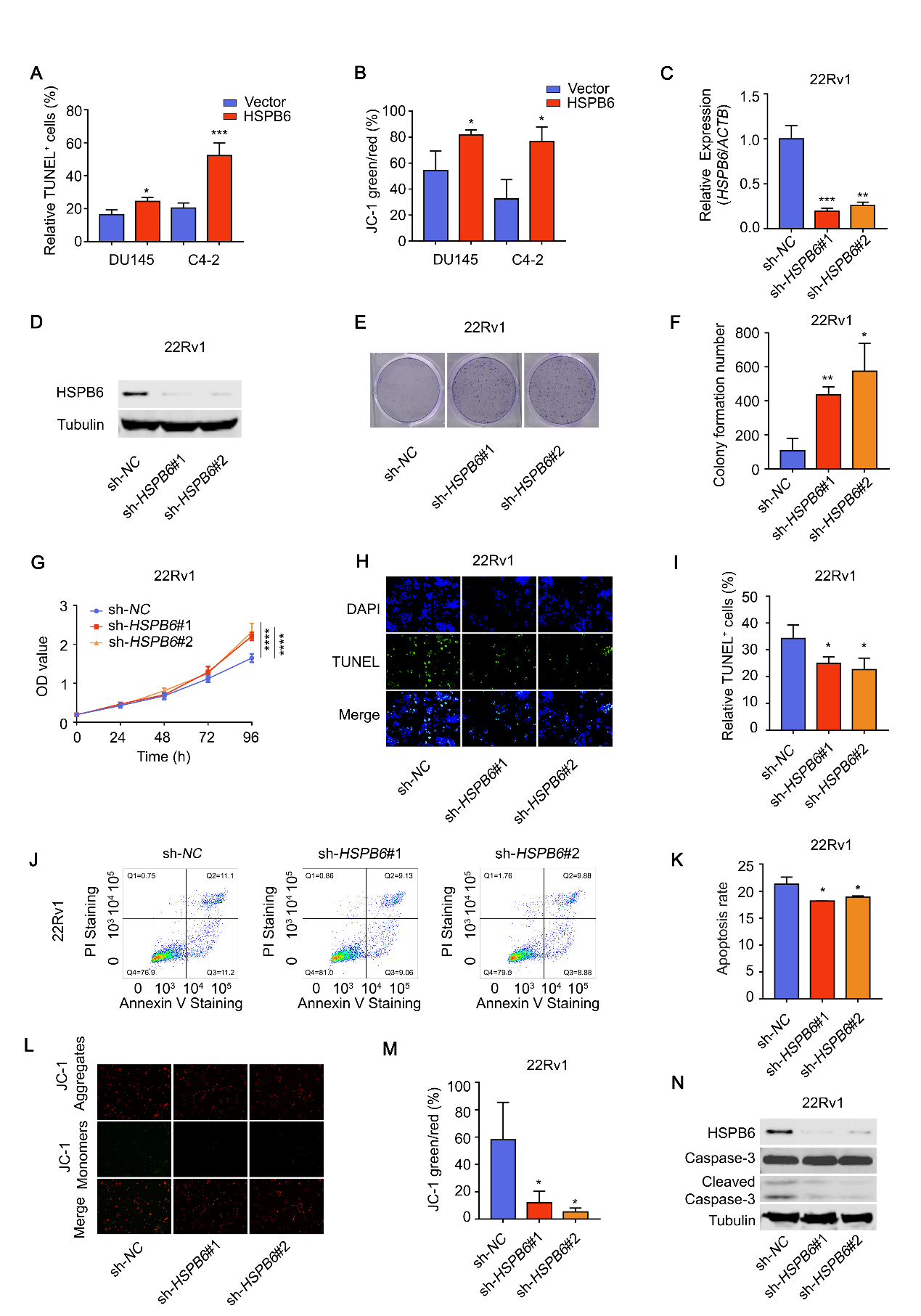


**Supplementary Figure 2, Supplemental to Figure 3.**

**A** The statistic of TUNEL level of prostate cancer cell lines (DU145 and C4-2) after overexpression of *HSPB6*. (*P* < 0.05 as "*", *P* < 0.001 as "***", unpaired t-test.) **B** The statistic of JC-1 level of prostate cancer cell lines (DU145 and C4-2) after overexpression of *HSPB6*. (*P* < 0.05 as "*", unpaired t-test.) **C, D** RT-qPCR (**C**) and Western blot analysis (**D**) showed the efficiency of knocking down *HSPB6* in 22Rv1. (*P* < 0.01 as "**", *P* < 0.001 as "***", unpaired t-test.) **E-G** Colony formation assays (**E, F**) and CCK8 (**G**) showed the proliferation ability of 22Rv1 after knockdown of *HSPB6*. (*P* < 0.05 as "*", *P* < 0.01 as "**", *P* < 0.0001 as "****", unpaired t-test, ANOVA.) **H-K** TUNEL (**H, I**) and flow cytometry (**J, K**) showed the apoptosis levels of 22Rv1 after the knockdown of *HSPB6*. (*P* < 0.05 as "*", unpaired t-test.) **L, M** Mitochondrial membrane potential levels of 22Rv1 after knockdown of *HSPB6* were shown by JC-1 staining. (*P* < 0.05 as "*", unpaired t-test.) **N** Western blot analysis showed that the expression of the apoptosis marker Cleaved Caspase-3 changed after the knockdown of *HSPB6*.


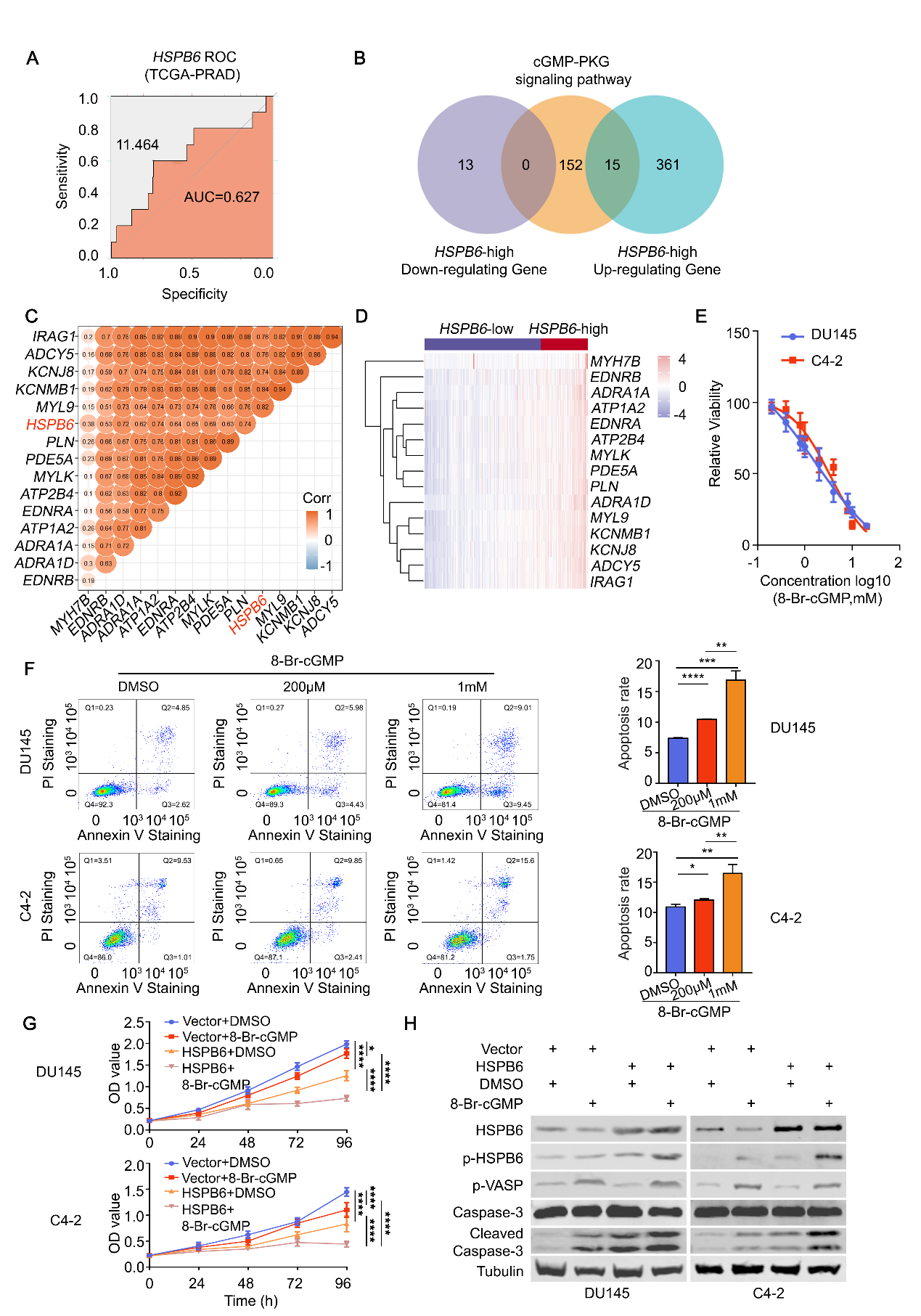


**Supplementary Figure 3, Supplemental to Figure 4.**

**A** The cut-off value was calculated using the ROC curve of *HSPB6* expression from TCGA database. **B** The Venn plot showed the intersection of the cGMP-PKG pathway genes with the differentially expressed genes between the *HSPB6* high and low-expression groups. **C** The correlation heatmap showed the correlation of *HSPB6* with key genes of the cGMP-PKG pathway. **D** The heat map showed the expression of key genes of the cGMP-PKG pathway in prostate cancer tissue from TCGA database. **E** The IC50 curve showed the sensitivity of DU145 and C4-2 to 8-Br-cGMP. **F** The flow cytometry showed the apoptosis levels of DU145 and C4-2 after supplementation with different concentrations of 8-Br-cGMP. (*P* < 0.05 as "*", *P* < 0.01 as "**", *P* < 0.001 as "***", *P* < 0.0001 as "****", unpaired t-test.) **G** CCK8 showed the proliferation ability of DU145 and C4-2 after overexpression HSPB6 and (or) supplementation with 8-Br-cGMP (200μM). (*P* < 0.05 as “*”, *P* < 0.0001 as “****”, ANOVA.) **H** Western blot analysis showed that the expression of p-HSPB6, p-VASP and Cleaved Caspase-3 after overexpression *HSPB6* and (or) supplementation with 8-Br-cGMP (200μM) (p-VASP as positive control).


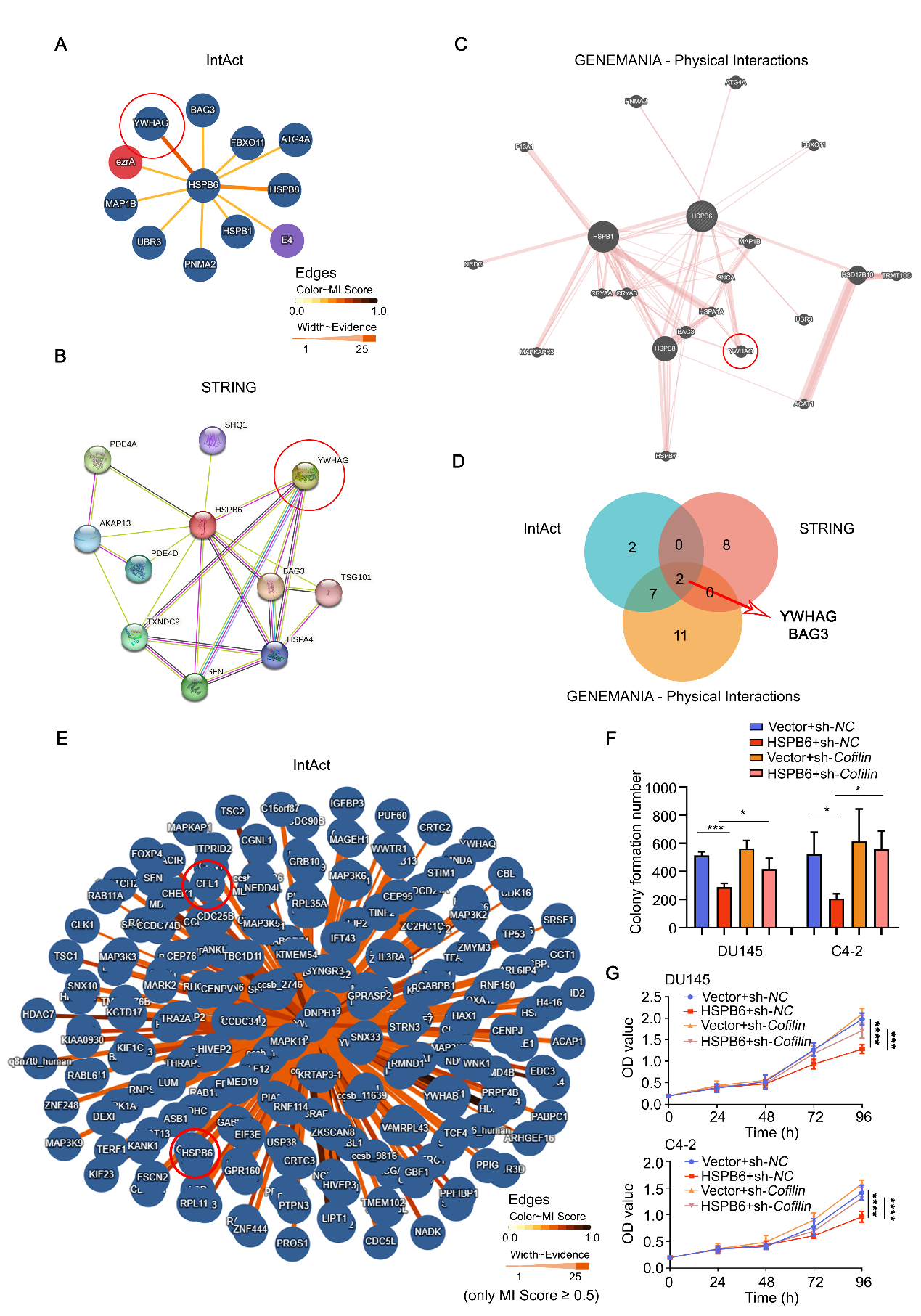


**Supplementary Figure 4, Supplemental to Figure 5.**

**A-C** Proteins that can interact with HSPB6 were analyzed through the IntAct (**A**), STRING (**B**), and GENEMANIA (**C**) databases. **D** The Venn plot showed the intersection of the proteins that can interact with HSPB6 were analyzed through the IntAct, STRING, and GENEMANIA database. **E** Proteins that can interact with YWHAG were analyzed using IntAct database. **F** The colony formation of prostate cancer cell lines (DU145 and C4-2) after knockdown *Cofilin* and (or) overexpression of *HSPB6*. (*P* < 0.05 as "*", *P* < 0.001 as "***", unpaired t-test.) **G** CCK8 showed the proliferation ability of DU145 and C4-2 after knockdown *Cofilin* and (or) overexpression of *HSPB6*. (*P* < 0.001 as “***”, *P* < 0.0001 as “****”, ANOVA.)


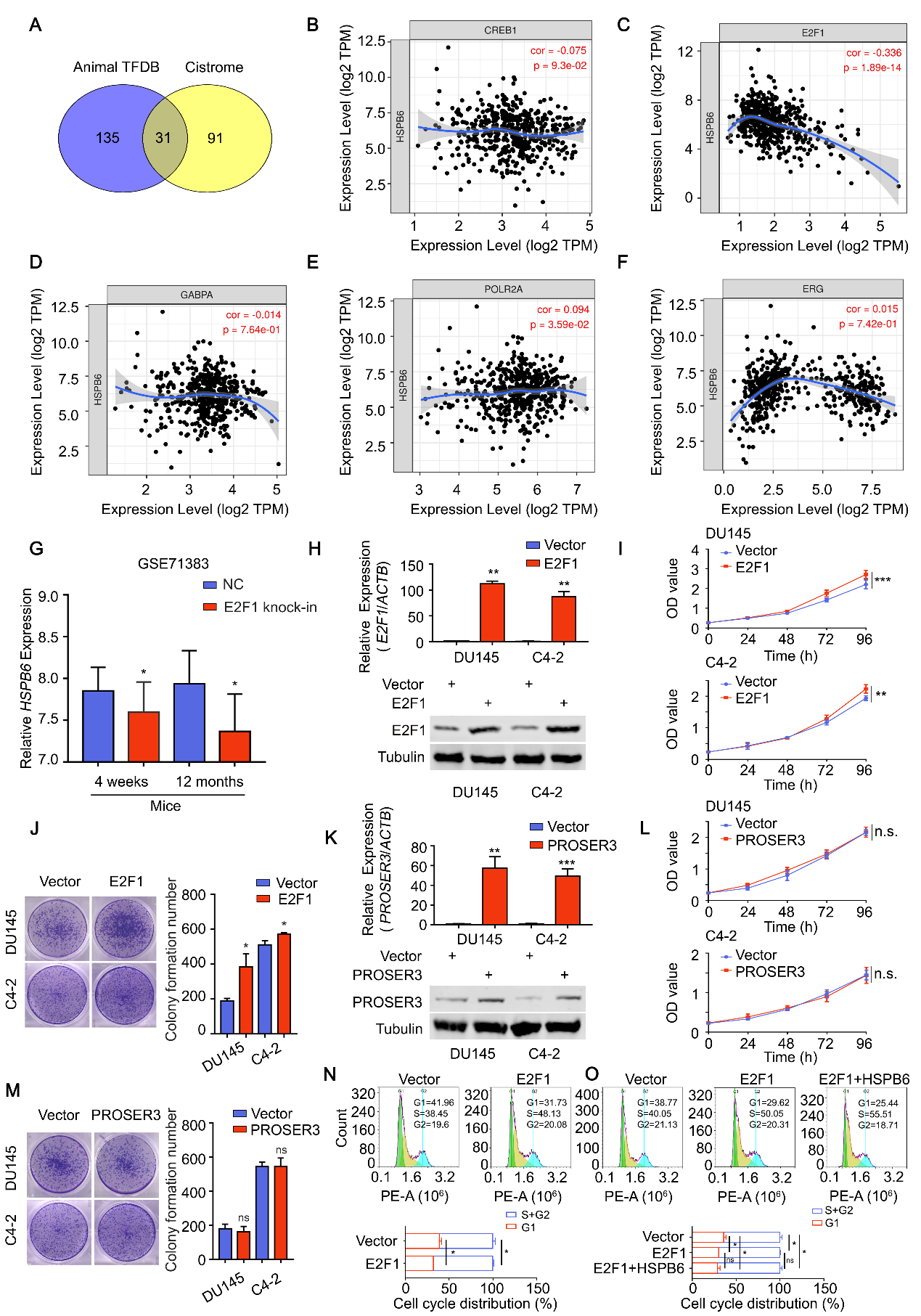


**Supplementary Figure 5, Supplemental to Figure 6, 7**

**A** The Venn plot showed the intersection of the transcription factors predicted from the Animal TFDB and Cistrome databases. **B-F** Analysis of the correlation between *HSPB6* and transcription factors genes (*CREB1*, *E2F1*, *GABPA*, *POLR2A* and *ERG*) from TCGA database. **G** The *HSPB6* expression after knocking in E2F1 from GEO database (GSE71383). **H** RT-qPCR and Western blot analysis showed the efficiency of overexpressing *E2F1* in DU145 and C4-2. (*P* < 0.01 as "**", unpaired t-test.) **I, J** CCK8 (**i**) and Colony formation assays (**j**) showed the proliferation ability of DU145 and C4-2 after overexpression of *E2F1*. (*P* < 0.05 as "*", *P* < 0.01 as "**", *P* < 0.001 as "***", unpaired t-test, ANOVA.) **K** RT-qPCR and Western blot analysis showed the efficiency of overexpressing *PROSER3* in DU145 and C4-2. (*P* < 0.01 as "**", *P* < 0.001 as "***", unpaired t-test.) **L, M** CCK8 (**L**) and Colony formation assays (**M**) showed the proliferation ability of DU145 and C4-2 after overexpression of *PROSER3*. (n.s. no specific, unpaired t-test, ANOVA.) **N** Flow cytometry showed the cell cycle distribution of DU145 and C4-2 after overexpression of *E2F1*. (*P* < 0.05 as "*", unpaired t-test.) **O** Flow cytometry showed the cell cycle distribution of DU145 and C4-2 after overexpression of *E2F1* and *HSPB6*. (n.s. no specific, *P* < 0.05 as "*", unpaired t-test.)


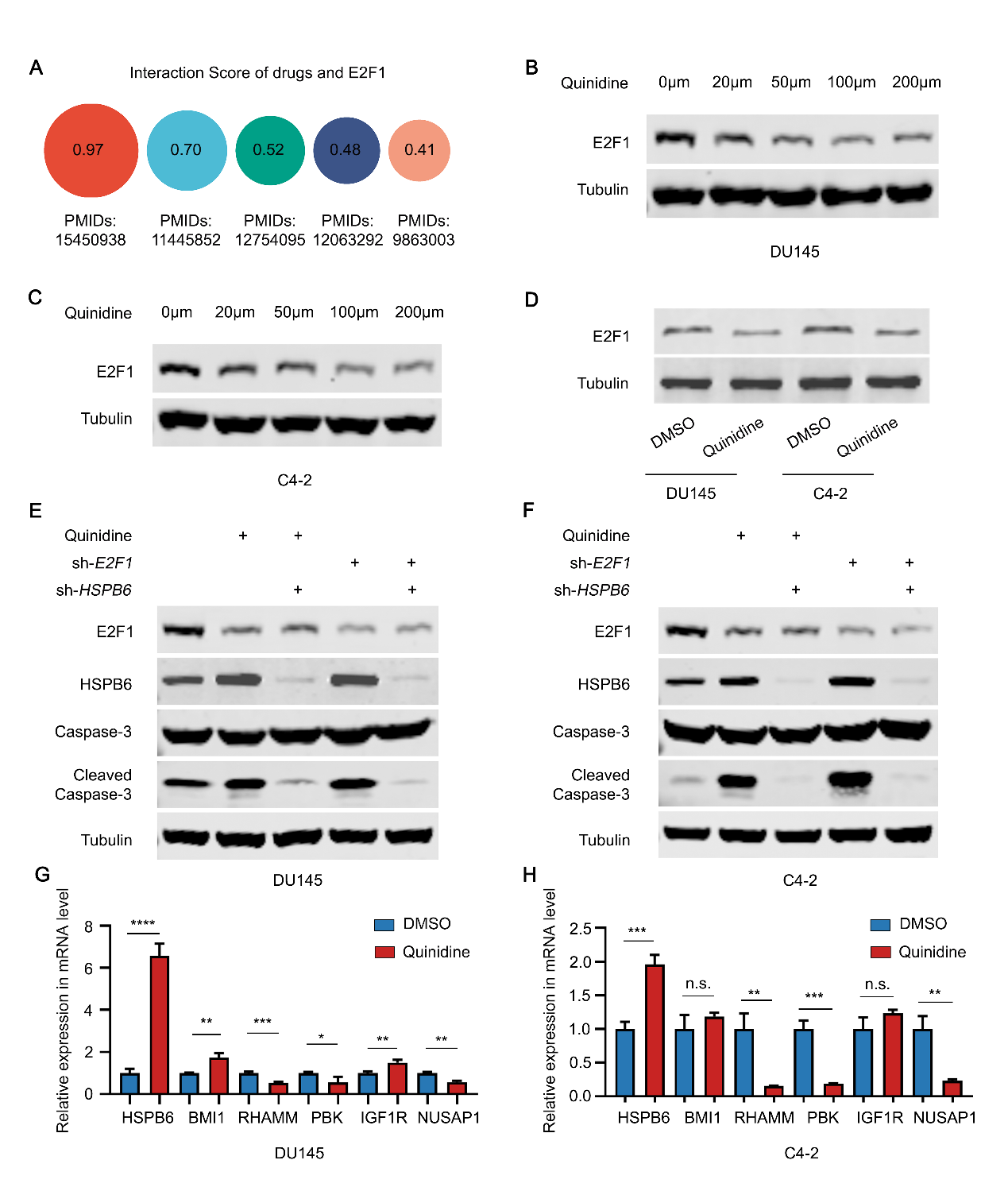


**Supplementary Figure 6, Supplemental to Figure 8**

**A** The interaction score of drugs and E2F1. **B, C** Western blot analysis showed the expression of E2F1 after supplementation with various concentrations of quinidine. **D** Western blot analysis showed the expression of E2F1 after supplementation with quinidine (100 μM). **E, F** Western blot analysis showed the expression of E2F1, HSPB6, Caspase-3 and Cleaved Caspase-3 after supplementation with quinidine (100 μM), sh-*E2F1* and (or) sh-*HSPB6*. **G, H** RT-qPCR were used to detect the expression of *HSPB6*, *BMI1*, *RHAMM*, *PBK*, *IGF1R* and *NUSAP1* after supplementation with quinidine (100 μM) (*P* < 0.05 as "*", *P* < 0.01 as "**", *P* < 0.001 as "***", n.s. means nonspecific, unpaired t-test.).


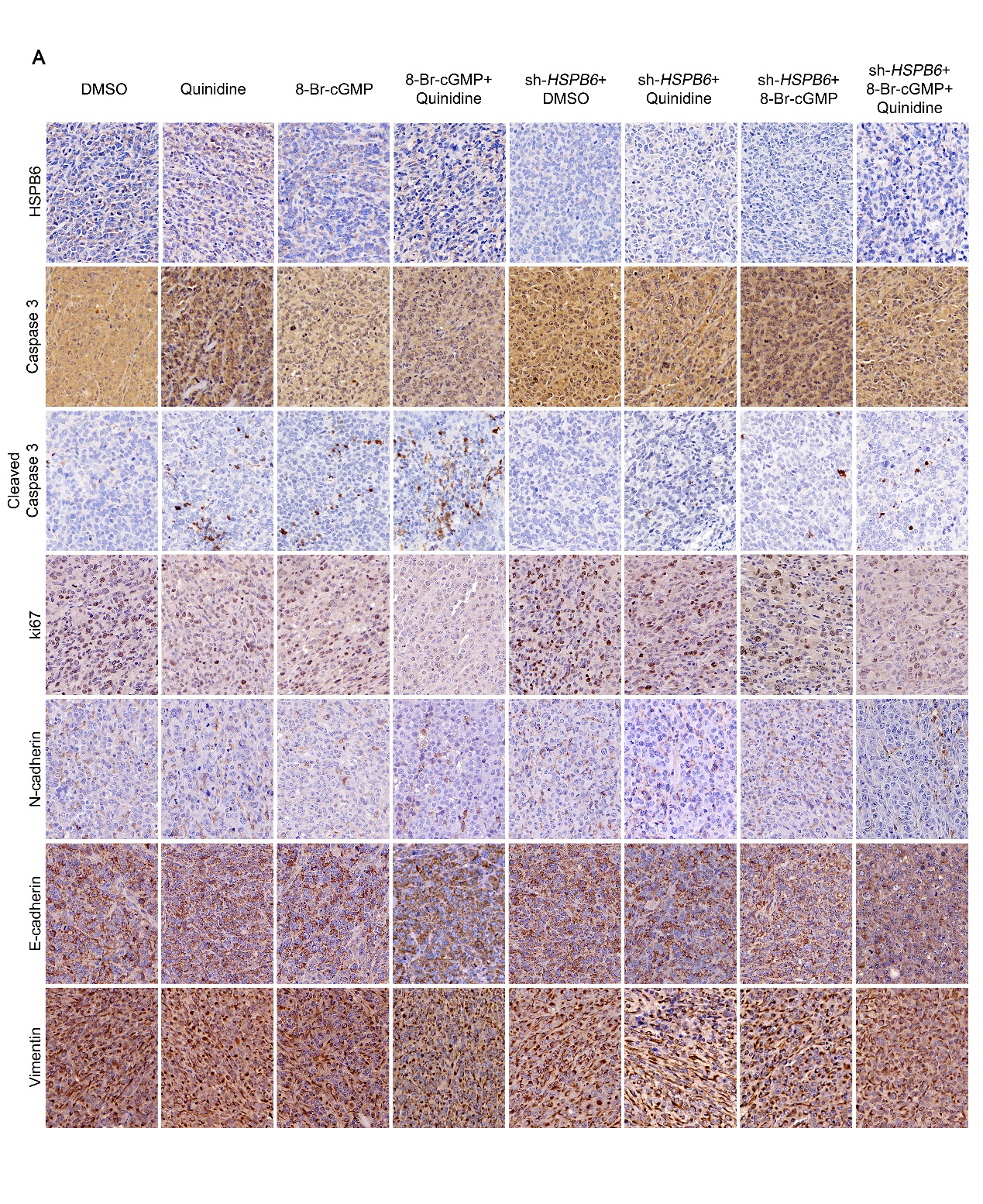


**Supplementary Figure 7, Supplemental to Figure 8**

**A** Expression of HSPB6, ki67, Caspase-3, Cleaved Caspase-3, N-cadherin, E-cadherin and Vimentin in xenografts of different groups by IHC method.


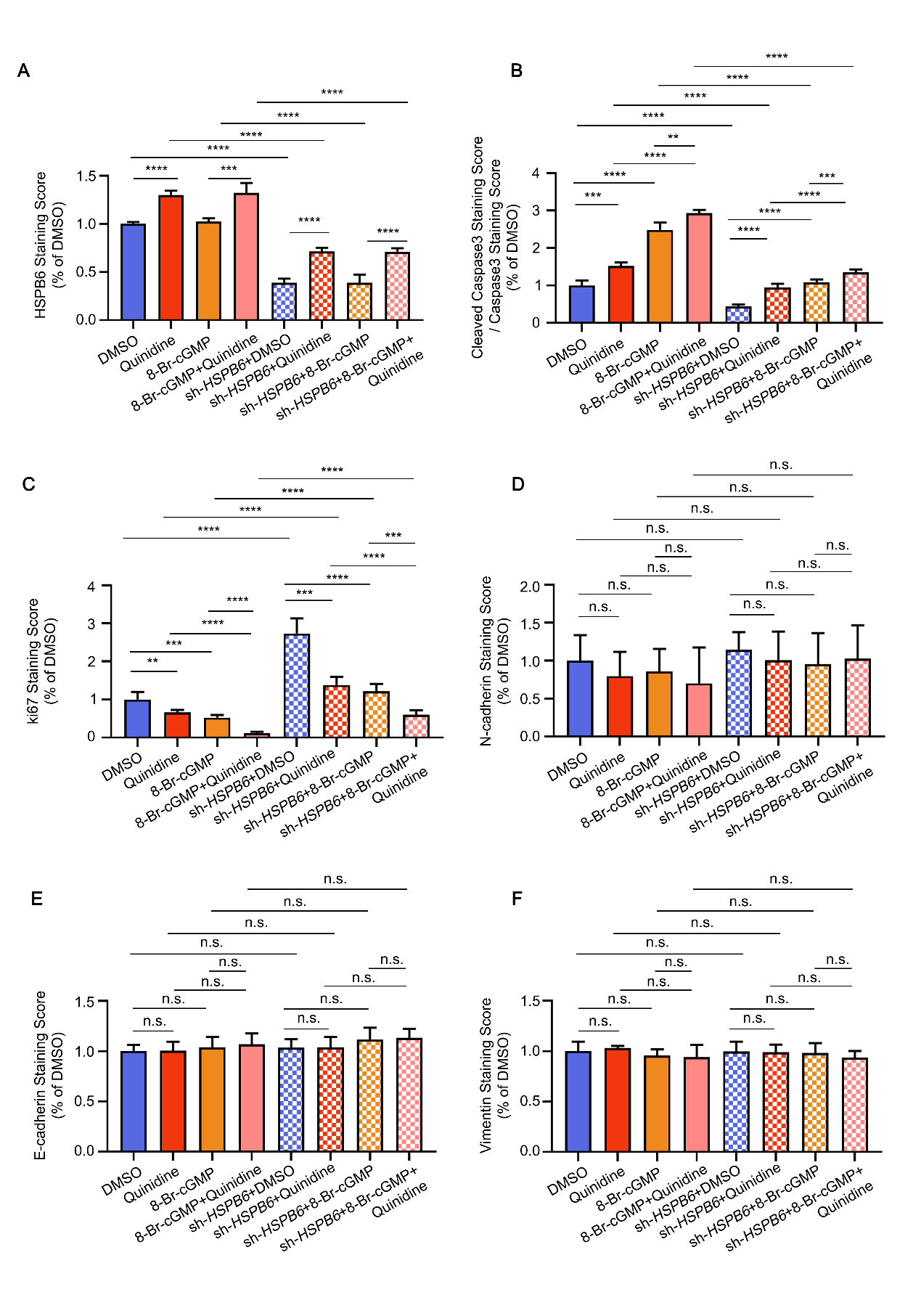


**Supplementary Figure 8, Supplemental to Figure 8**

**A-F** Expression of HSPB6, ki67, Caspase-3, Cleaved Caspase-3, N-cadherin, E-cadherin and Vimentin in xenografts of different groups by IHC method. All data expressed as mean ± S.E.M. (*P* < 0.01 as “**”, *P* < 0.001 as “***”, *P* < 0.0001 as “****”, n.s. means nonspecific, unpaired t-test.)

**Supplementary Table S1. Cell lines, antibodies and other reagents and resources**

| **REAGENT or RESOURCE** | **SOURCE** | **IDENTIFIER** |
| --- | --- | --- |
| **Cell Lines** | | |
| Human: C4-2 | Bank of the Chinese Academy of Sciences | CRL-1435 |
| Human: PC-3 | Bank of the Chinese Academy of Sciences | HTB-81 |
| Human: HEK293T | Bank of the Chinese Academy of Sciences | CRL-1740 |
| Human: 22Rv1 | Bank of the Chinese Academy of Sciences | CRL-2876 |
| Human: LNCaP | Bank of the Chinese Academy of Sciences | CRL-1740 |
| Human: VCaP | Bank of the Chinese Academy of Sciences | CRL-2876 |
| Human: DU145 | Bank of the Chinese Academy of Sciences | HTB-81 |
|  | | |
| **Antibodies** | | |
| Mouse monoclonal anti-beta Actin  (WB: 1 µg/ml) | Abcam | Cat# ab8226 |
| Rabbit polyclonal anti-HSPB6  (WB: 1/500, IHC/IF: 1/50) | Solarbio | Cat# K009075P |
| Rabbit polyclonal anti-phospho–  Hsp20  (WB: 1/1000) | Abcam | Cat# ab58522 |
| Mouse monoclonal anti-Caspase-3  (WB: 1/200) | Santa Cruz | Cat# sc-56053 |
| Rabbit monoclonal anti-Cofilin  (WB: 1/1000) | Cell Signaling Technology | Cat# 5175 |
| Rabbit monoclonal anti-Phospho-  Cofilin  (WB: 1/1000) | Cell Signaling Technology | Cat# 3313 |
| Rabbit monoclonal anti-YWHAG  (WB:1/1000) | Cell Signaling Technology | Cat# 5522 |
| Mouse monoclonal anti- E2F1  (WB:1/200) | Santa Cruz | Cat# sc-251 |
| Rabbit polyclonal anti-PROSER3  (WB: 1 µg/mL) | OriGene | Cat# TA339978 |
| Dylight 800, Goat Anti-Rabbit IgG  (WB: 1/5000) | Abbkine | Cat# A23920 |
| Dylight 800, Goat Anti-Mouse IgG  (WB: 1/10000) | Abbkine | Cat# A23910 |
| Rabbit polyclonal anti-phospho-  VASP  (WB:1/1000) | Abcam | Cat# ab194747 |
| Rabbit polyclonal anti-Cleaved-  Caspase-3  (IHC: 1/500) | Servicebio | Cat# GB11532-50 |
| Rabbit polyclonal anti-Cofilin  (IF: 1/650) | Servicebio | Cat# GB112496-100 |
| Rabbit polyclonal anti-YWHAG  (IF: 1/50) | Solarbio | Cat# K008882P |
| Rabbit monoclonal anti-N-Cadherin  (WB: 1/1000) | Cell Signaling Technology | Cat # 13116 |
| Rabbit monoclonal anti-E-Cadherin  (WB: 1/1000) | Cell Signaling Technology | Cat # 3195 |
| Rabbit monoclonal anti-Vimentin  (WB: 1/1000) | Cell Signaling Technology | Cat # 5741 |
| Rabbit monoclonal anti-PCNA  (WB: 1/1000) | Cell Signaling Technology | Cat # 13110 |
| Rabbit polyclonal anti-Caspase-3  (IHC: 1/100) | Servicebio | Cat# GB11009-100 |
| Mouse monoclonal anti- E-Cadherin  (IHC: 1/500) | Servicebio | Cat# GB12083-100 |
| Mouse monoclonal anti- Ki67  (IHC: 1/300) | Servicebio | Cat# GB121141-100 |
| Mouse monoclonal anti- N-Cadherin  (IHC: 1/500) | Servicebio | Cat# GB12135-100 |
| Rabbit polyclonal anti- Vimentin  (IHC: 1/200) | Servicebio | Cat# GB11192-100 |
|  |  |  |
| **Bacterial and Virus Strains** |  |  |
| *E. coli* DH5α | Thermo Fisher | Cat#18258012 |
| *E. coli* BL21 | Thermo Fisher | Cat# C600003 |
|  | | |
| **Chemicals, Peptides, and**  **Recombinant Proteins** |  |  |
| Quinidine | MedChemExpress | Cat# HY-B1751 |
| 8-Br-cGMP | Abcam | Cat# ab141449 |
| Polybrene  (Hexadimethrine Bromide) | Beyotime | Cat# C0351 |

**Supplementary Table S2. Sequence information of shRNAs**

| **Gene** | **Sequence (5’-3’)** |
| --- | --- |
| Sh-control | 5′- -3′CAACAAGATGAAGAGCACCAA |
| Sh-*HSPB6* -#1 | 5′- -3′GAGGAAATTGCTGTCAAGGTG |
| Sh-*HSPB6* -#2 | 5′- -3′GAAATTGCTGTCAAGGTGGTG |
| Sh-*E2F1*-#1 | 5′- -3′CATCCAGCTCATTGCCAAGAA |
| Sh-*E2F1*-#2 | 5′- -3′CTACTCAGCCTGGAGCAAGAA |

**Supplementary Table S3. Sequence information of primers for RT-qPCR**

| **Species** | **Gene** | **Forward (5’-3’)** | **Reverse (5’-3’)** |
| --- | --- | --- | --- |
| Human | *ACTB* | CTCCATCCTGGCCTCGCTGT | GCTGTCACCTTCACCGTTCC |
| Human | *HSPB6* | TTTCGGTGCTGCTAGACGTGAAG | GCGACGAATCCGTGCTCATCC |
| Human | *E2F1* | TGCCAAGAAGTCCAAGAACCACATC | TGTCGGAGGTCCTGGGTCAAC |
| Human | *PROSER3* | CCACTACTGGCCATCCCAGA | GCCAGGACTCCTCAAACAGC |
| Human | *IGF1R* | GCGATTGCTGGGTGTGGTGTC | GTTGAGGTATGCCATGCCGTCTG |
| Human | *BMI1* | TGGACTGACAAATGCTGGAGAACTG | GTTACCGCTGGGGCTGTTGC |
| Human | *NUSAP1* | TGAGCATAAGCGTTCACTGACCAAG | GAGTCTGCGTTGCCTCAGTTGTC |
| Human | *PBK* | CCCAAAGAAGCTGTGGAGGAGAATG | GCCTAGTTCCCAACGCTGCATAG |
| Human | *RHAMM* | ACCTTGCCTGCTTCAGCTAGAAAAG | TCCCTTAGTGCAGCATTTAGCCTTG |
