# Supplementary material for: 8-Br-cGMP activates HSPB6 and increases the antineoplastic activity of quinidine in prostate cancer
Source: Cell Death Discov. 2024 Feb 19;10:90. doi: 10.1038/s41420-024-01853-3 (PMC10876707; doi:10.1038/s41420-024-01853-3)
Supplement: Supplementary file 3 — Full and uncropped western blots [file 41420_2024_1853_MOESM3_ESM.pdf]

Figure 1

g

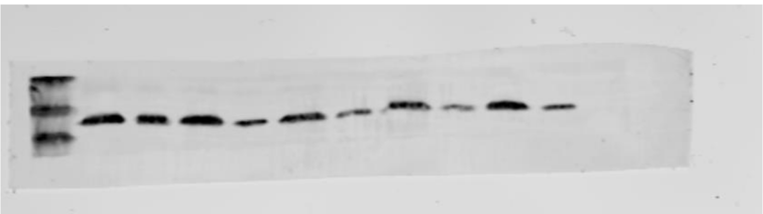

HSPB6

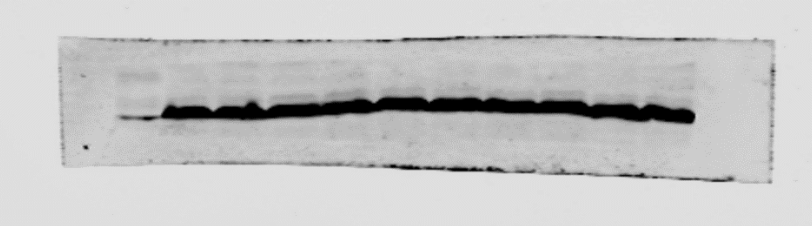

Tubulin

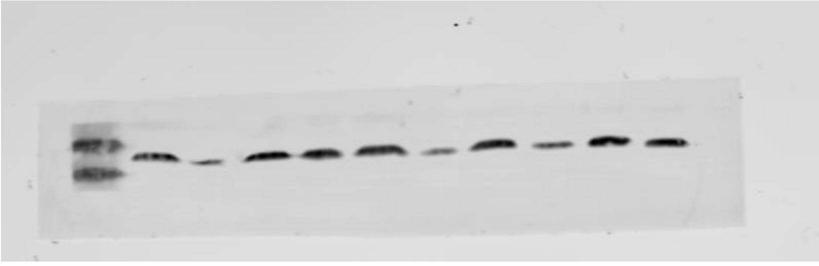

HSPB6

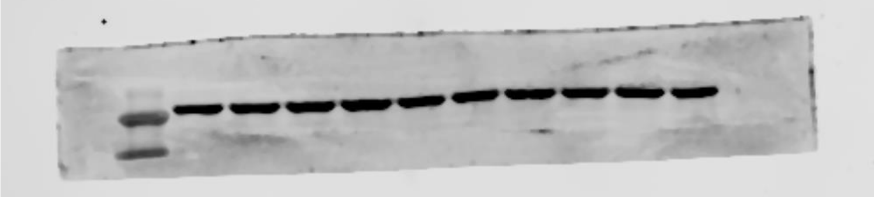

Tubulin

Figure 3

c

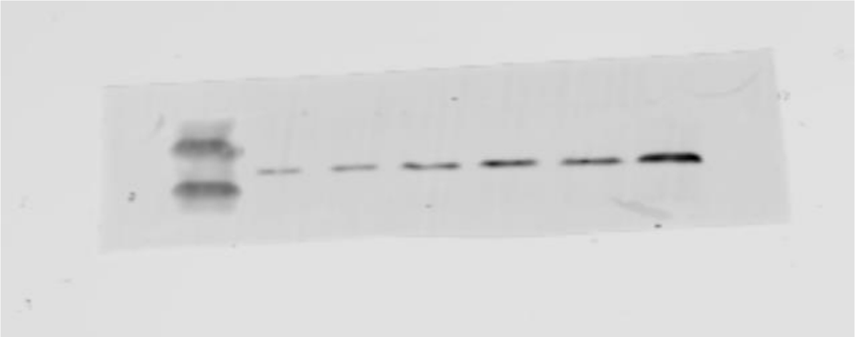

HSPB6

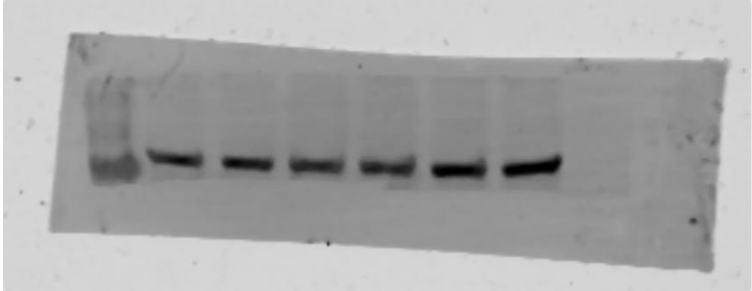

Tubulin

e

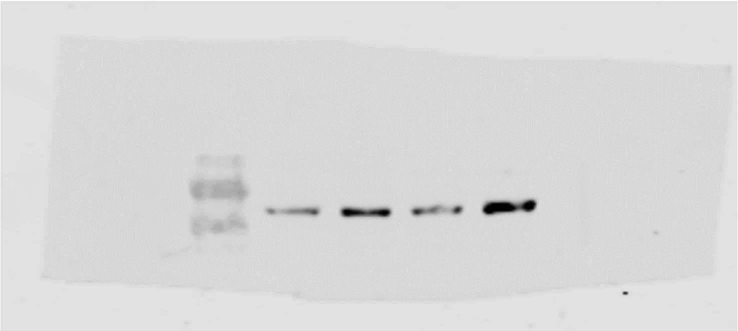

HSPB6

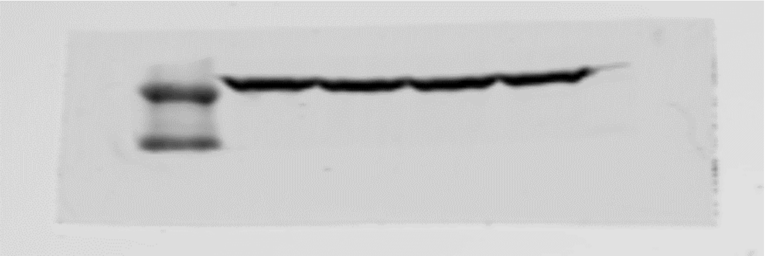

Tubulin

m

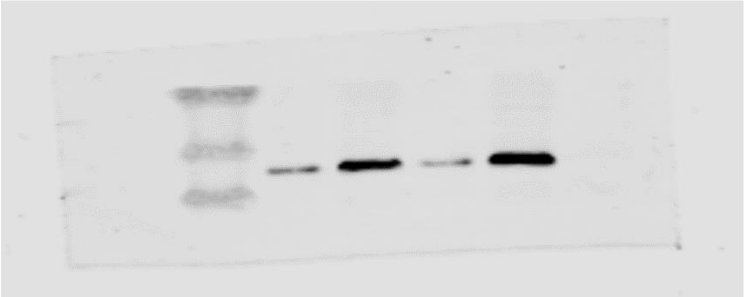

HSPB6

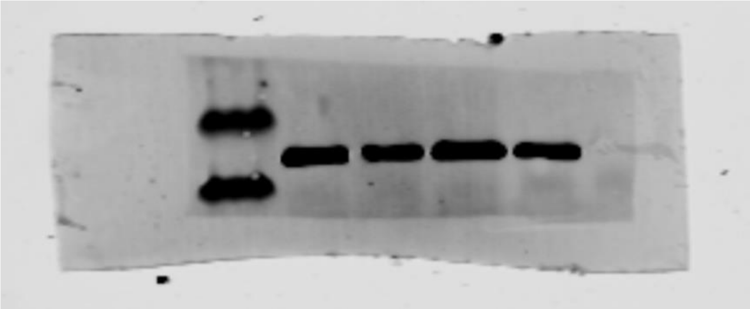

Caspase-3

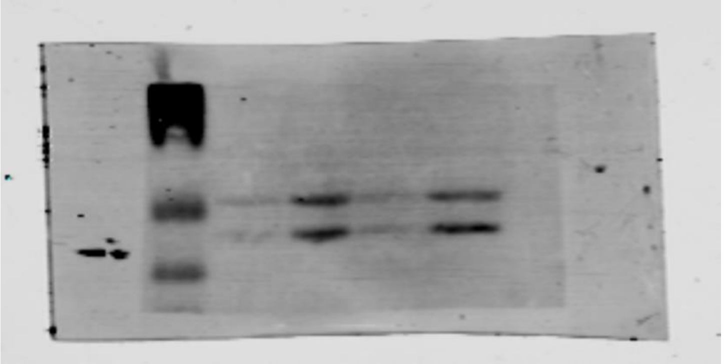

Cleaved Caspase-3

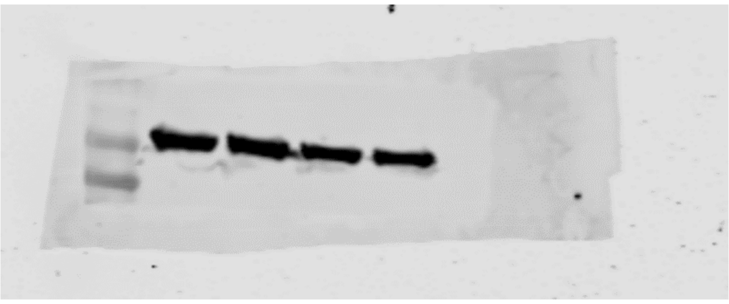

Tubulin

Figure 4

d

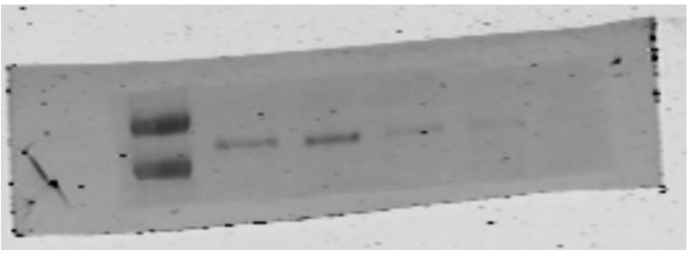

HSPB6 DU145

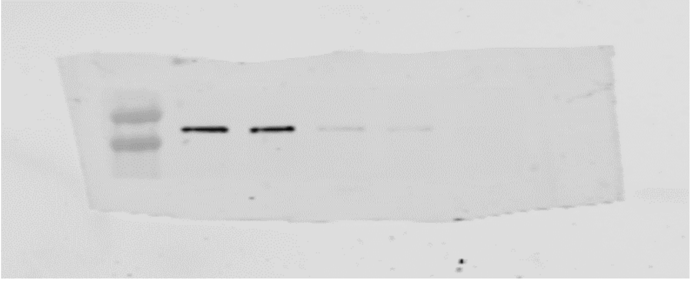

HSPB6 C4-2

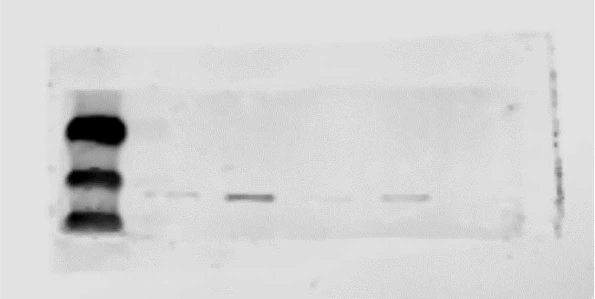

p-HSPB6 DU145

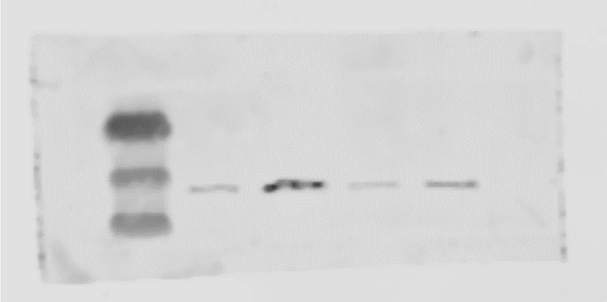

p-HSPB6 C4-2

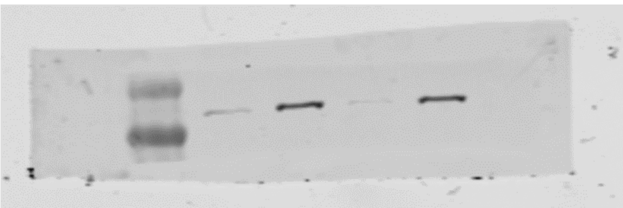

p-VASP DU145

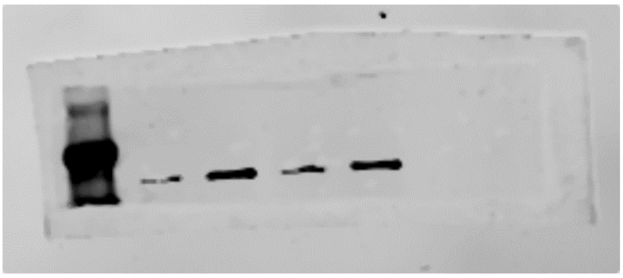

p-VASP C4-2

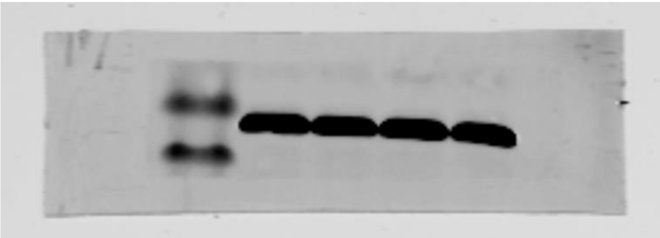

Caspase-3 DU145

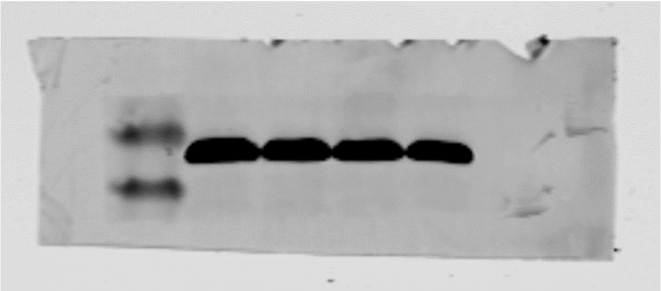

Caspase-3 C4-2

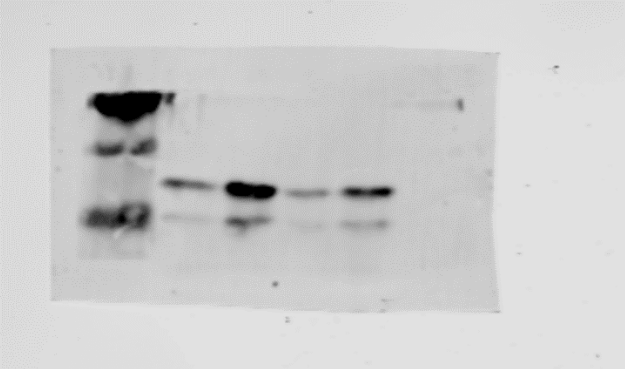

Cleaved Caspase-3 DU145

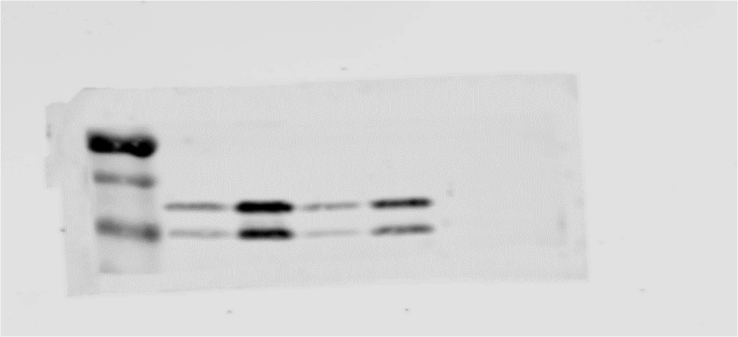

Cleaved Caspase-3 C4-2

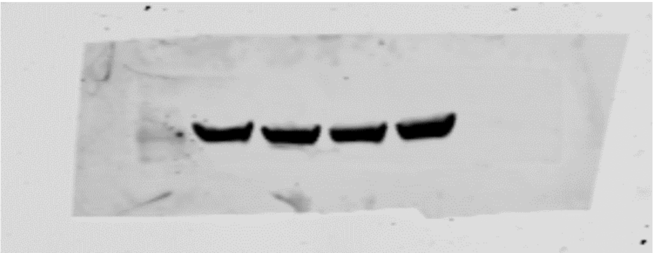

Tubulin DU145

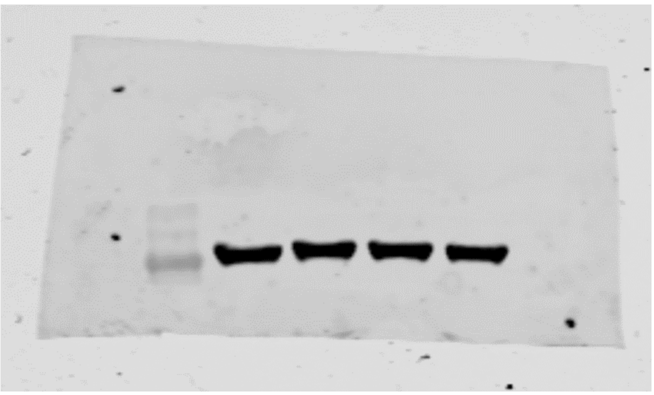

Tubulin C4-2

Figure 5

d

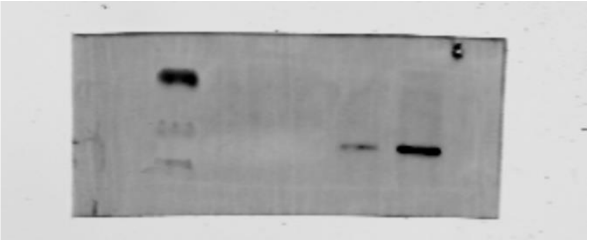

IP: p-HSPB6 DU145

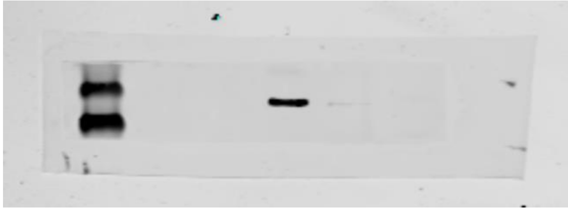

IP: p-Cofilin DU145

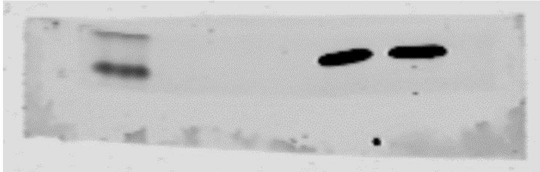

YWHAG DU145

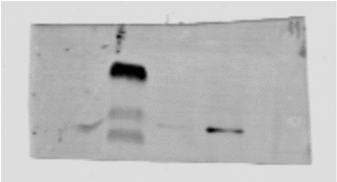

Input: p-HSPB6 DU145

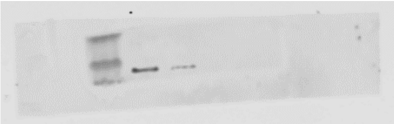

Input: p- Cofilin DU145

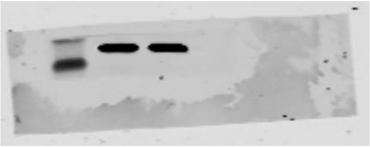

Input: YWHAG DU145

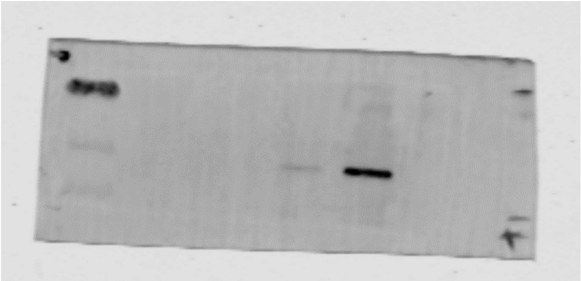

IP: p-HSPB6 C4-2

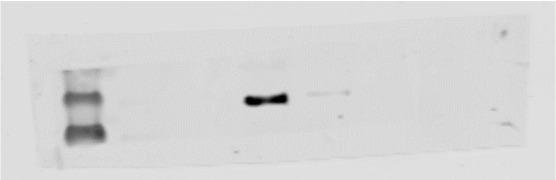

IP: Cofilin C4-2

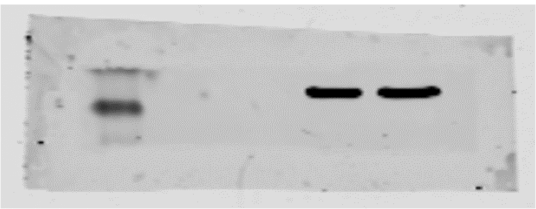

YWHAG C4-2

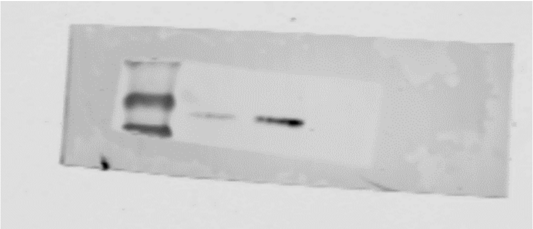

Input: p-HSPB6 C4-2

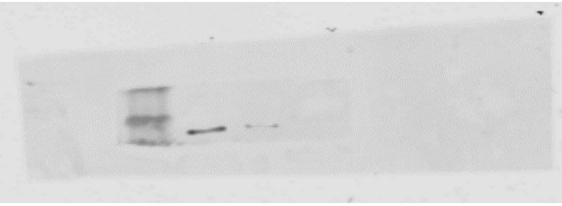

Input: p- Cofilin C4-2

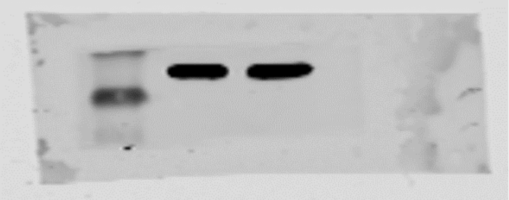

Input: YWHAG C4-2

e

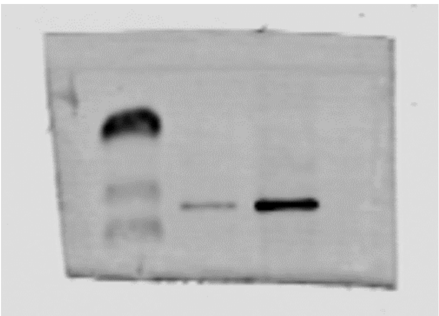

HSPB6 DU145

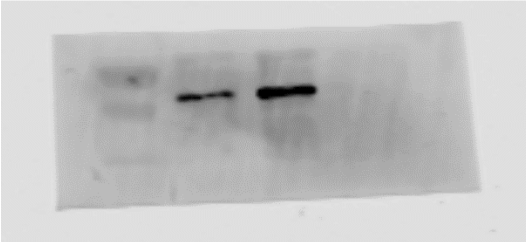

HSPB6 C4-2

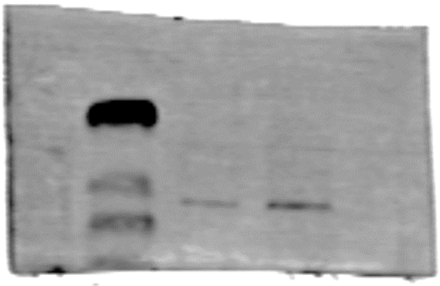

p-HSPB6 DU145

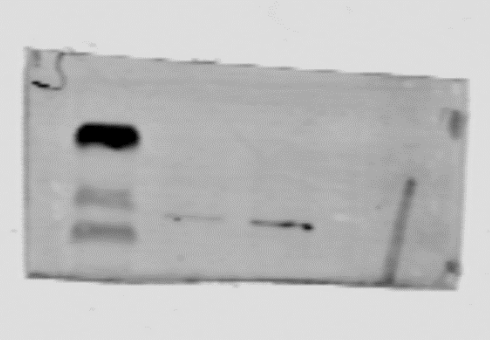

p-HSPB6 C4-2

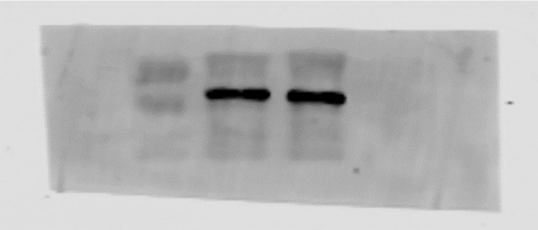

Cofilin DU145

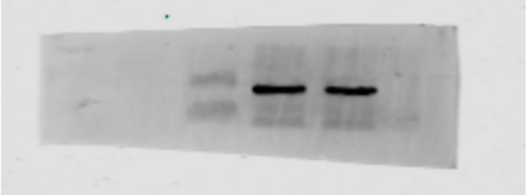

Cofilin C4-2

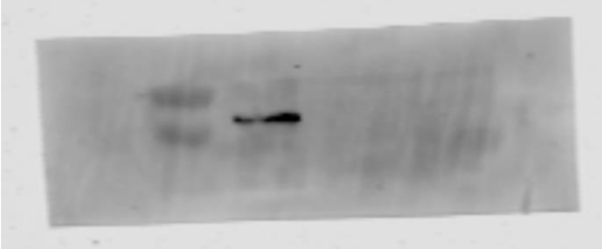

p-Cofilin DU145

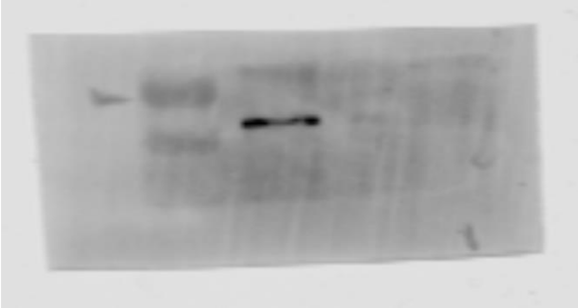

p-Cofilin C4-2

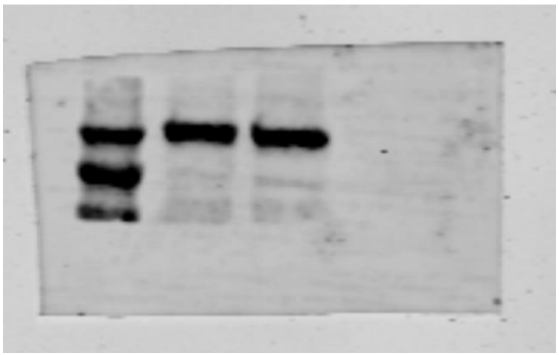

Tubulin DU145

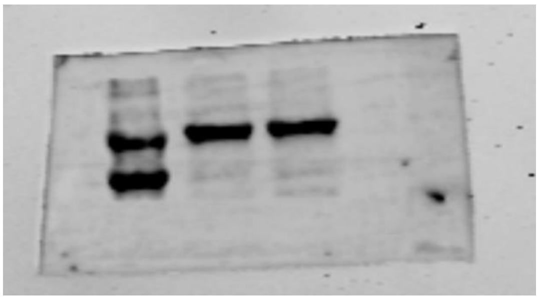

Tubulin C4-2

Figure 7

b

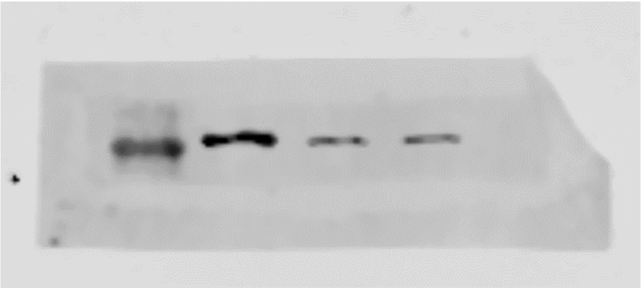

E2F1 DU145

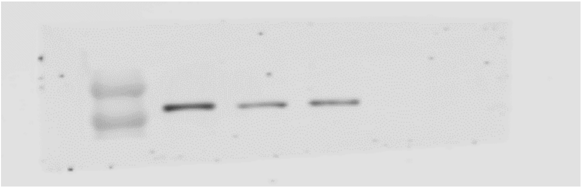

PROSER3 DU145

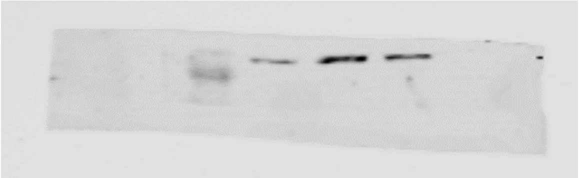

HSPB6 DU145

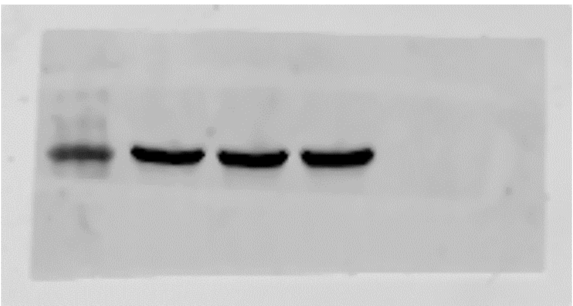

Tubulin DU145

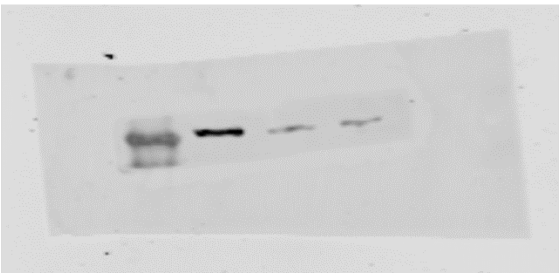

E2F1 C4-2

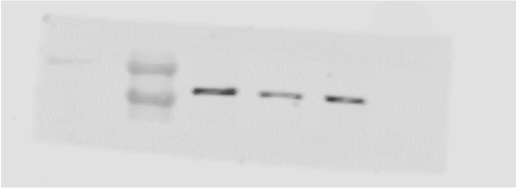

PROSER3 C4-2

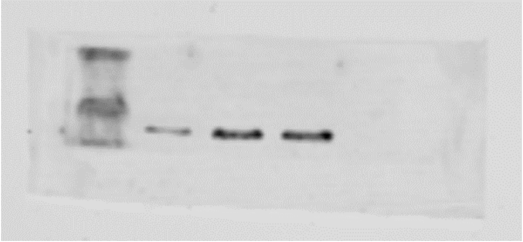

HSPB6 C4-2

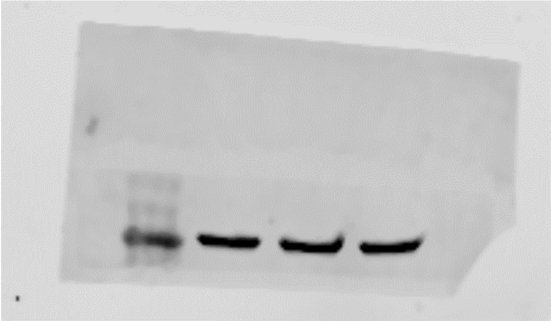

Tubulin C4-2

d

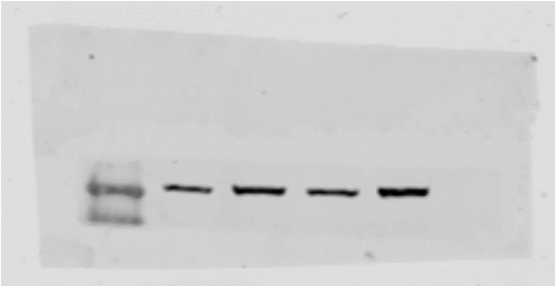

E2F1

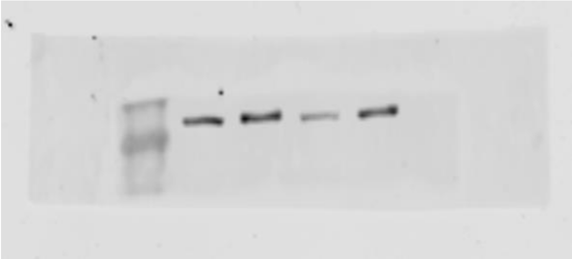

PROSER3

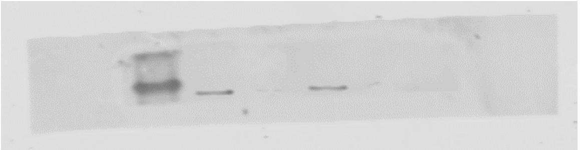

HSPB6

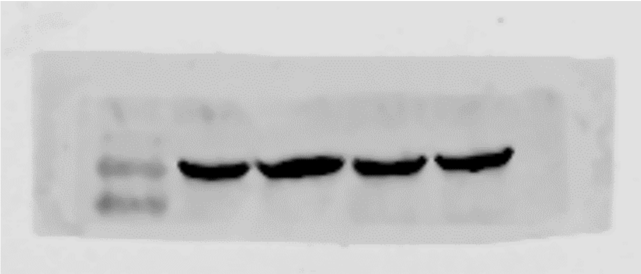

Tubulin

i

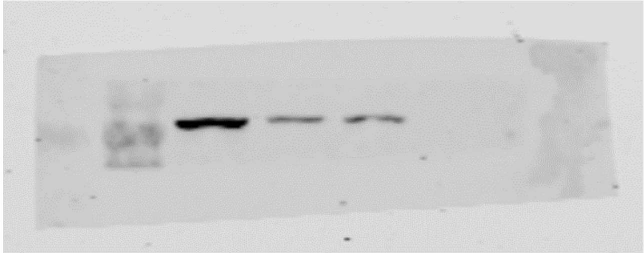

E2F1 DU145

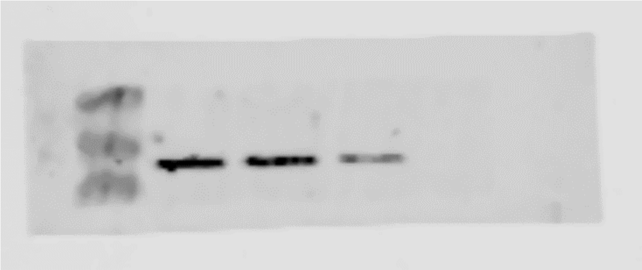

HSPB6 DU145

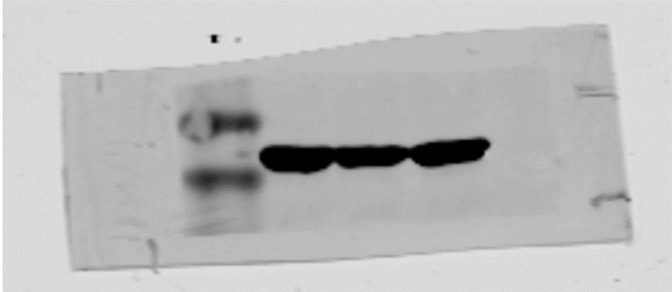

Caspase-3 DU145

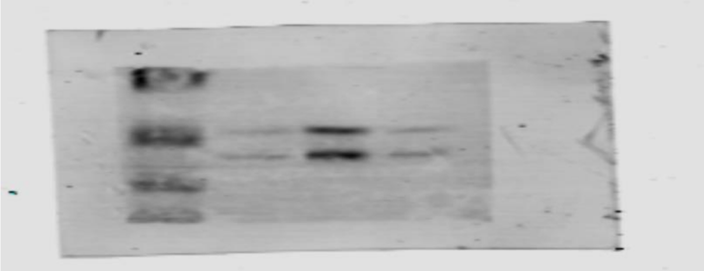

Cleaved Caspase-3 DU145

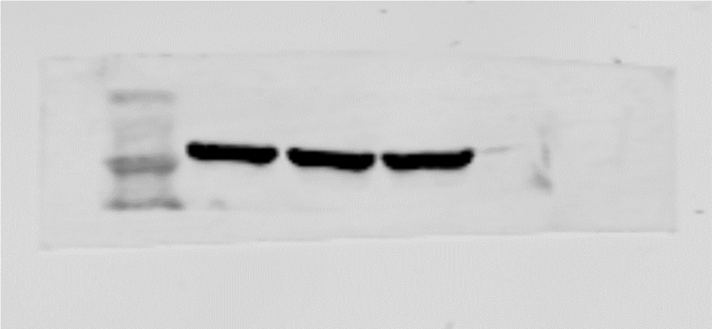

Tubulin DU145

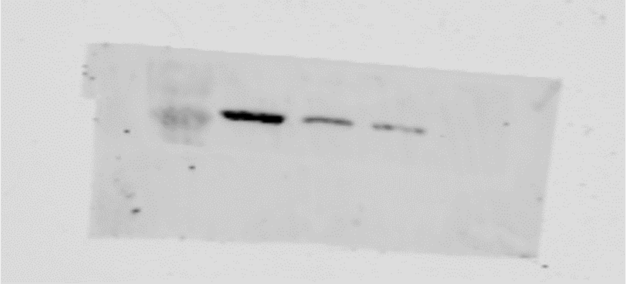

E2F1 C4-2

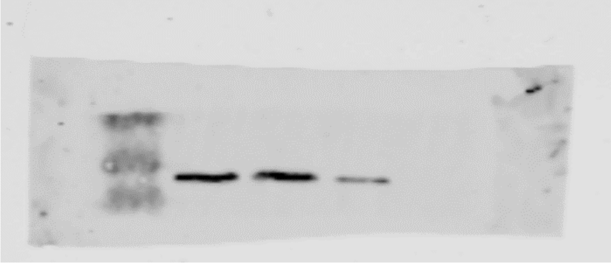

HSPB6 C4-2

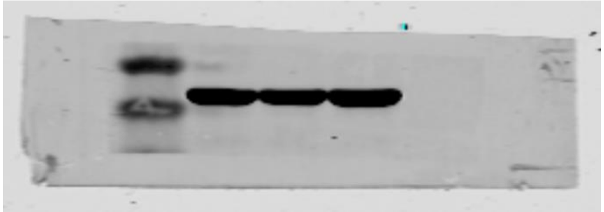

Caspase-3 C4-2

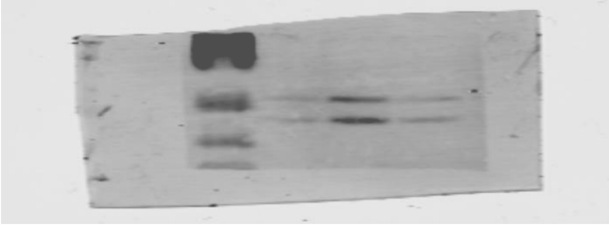

Cleaved Caspase-3 C4-2

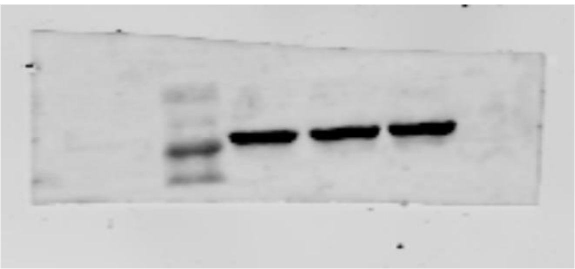

Tubulin C4-2

Supplementary Figure 1

h

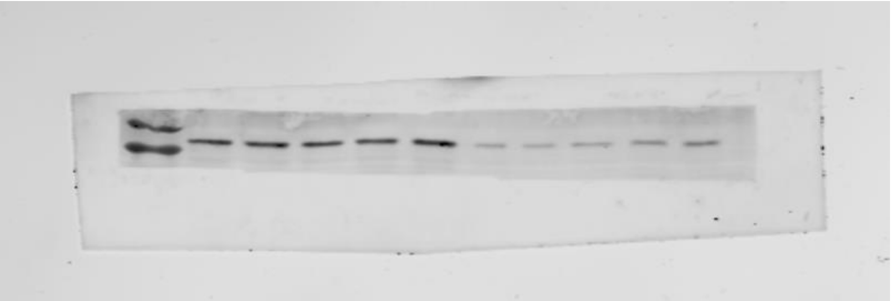

HSPB6

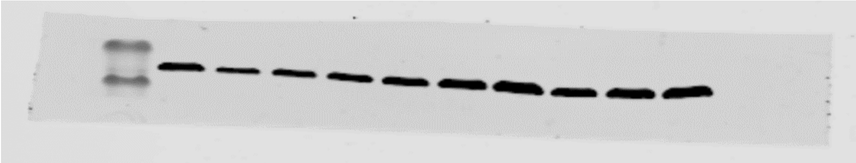

PCNA

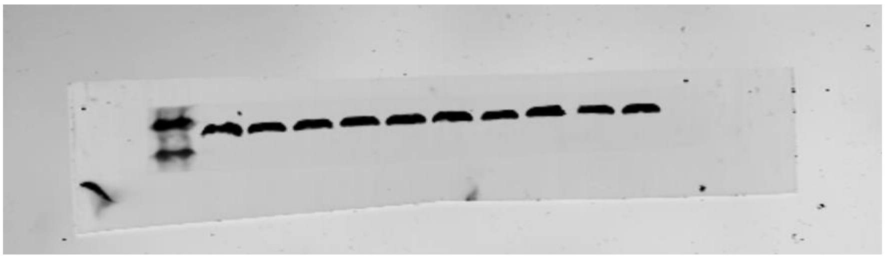

Caspase-3

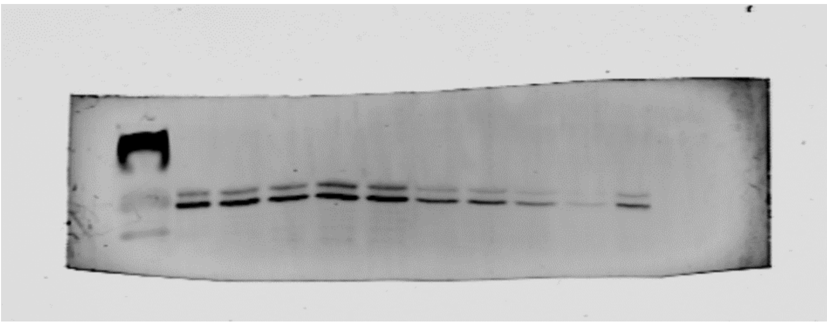

Cleaved Caspase-3

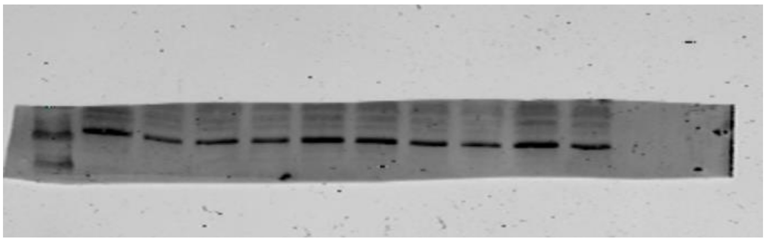

N-cadherin

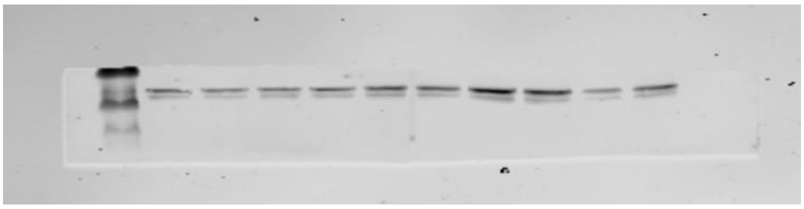

E-cadherin

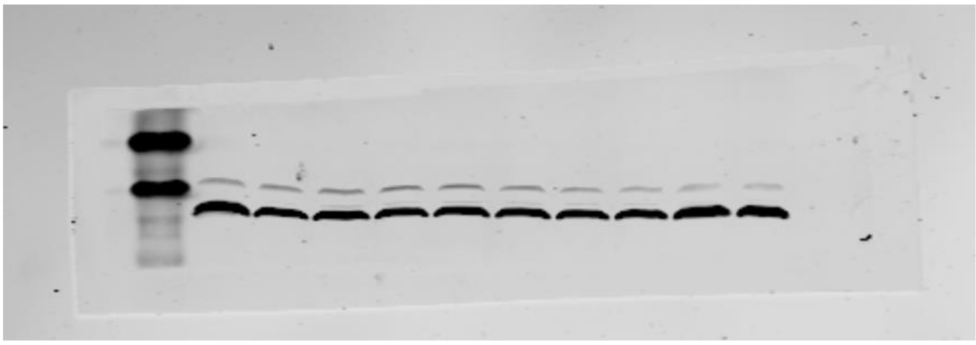

Vimentin

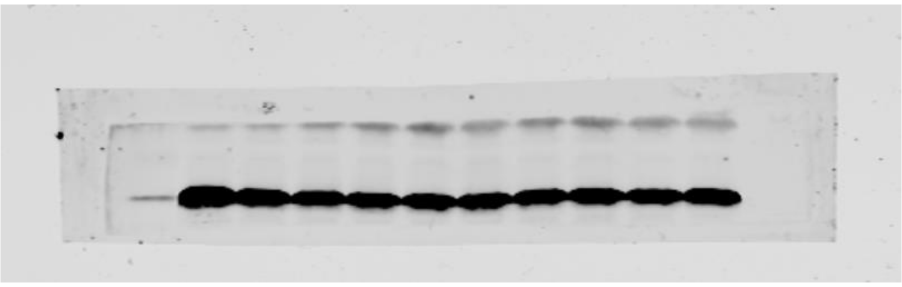

Tubulin

Supplementary Figure 2

d

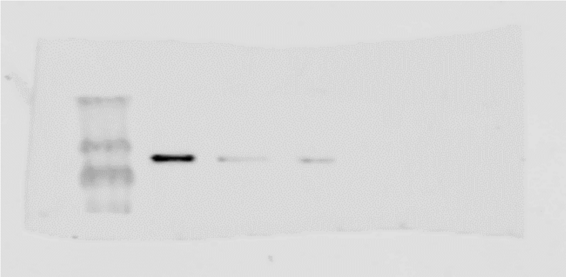

HSPB6

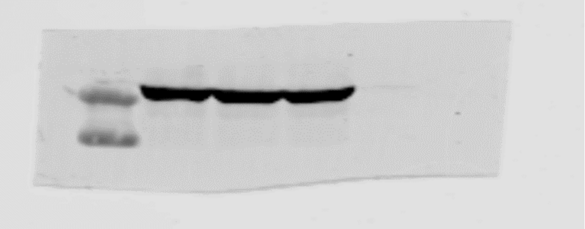

Tubulin

n

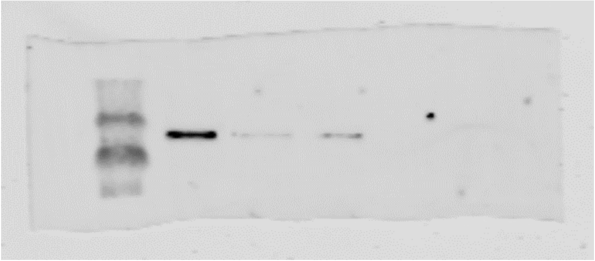

HSPB6

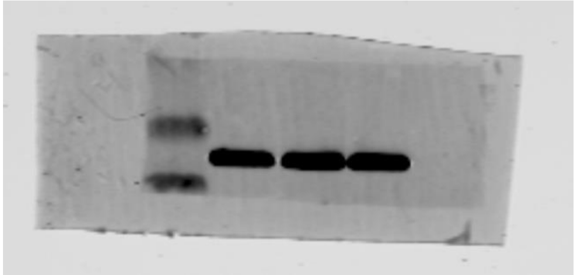

Caspase-3

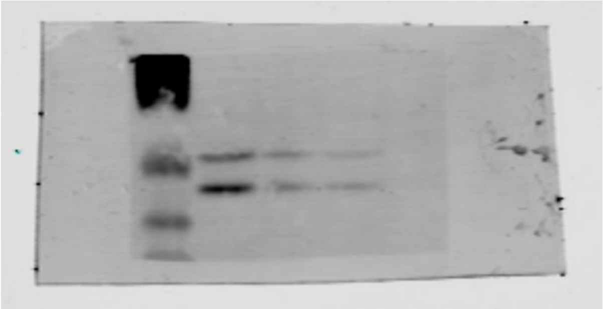

Cleaved Caspase-3

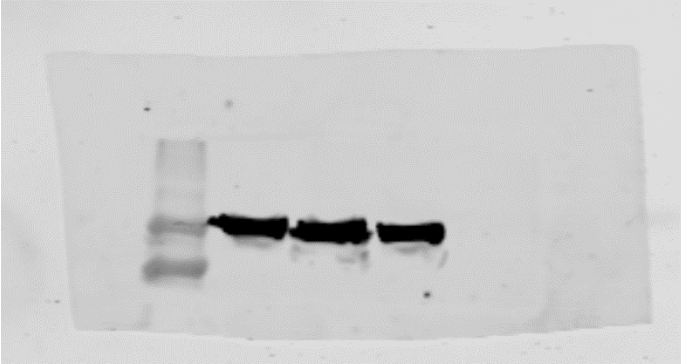

Tubulin

Supplementary Figure 3

h

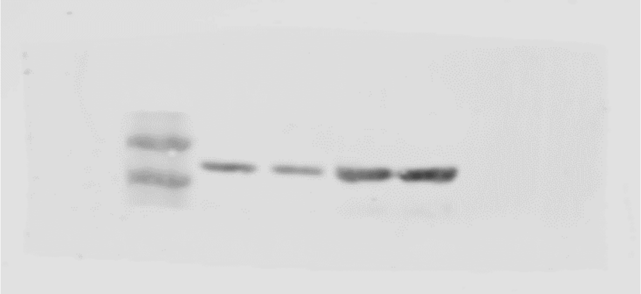

HSPB6 DU145

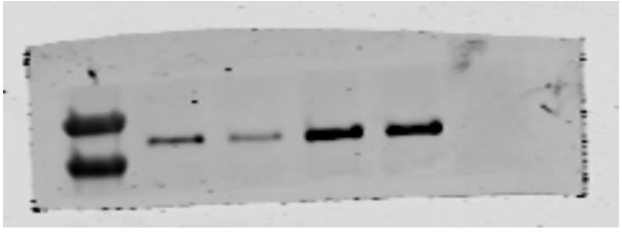

HSPB6 C4-2

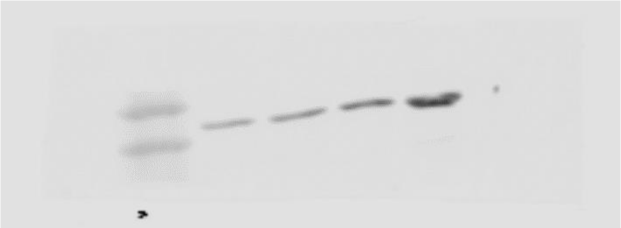

p-HSPB6 DU145

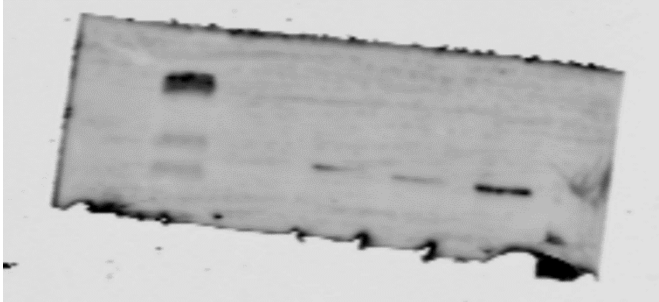

p-HSPB6 C4-2

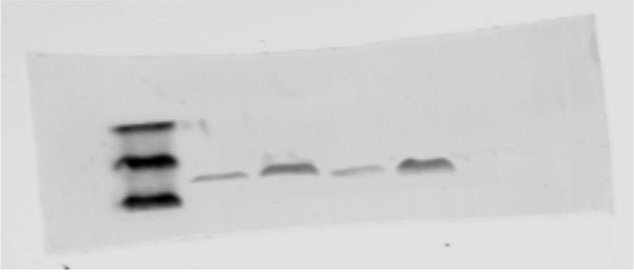

p-VASP DU145

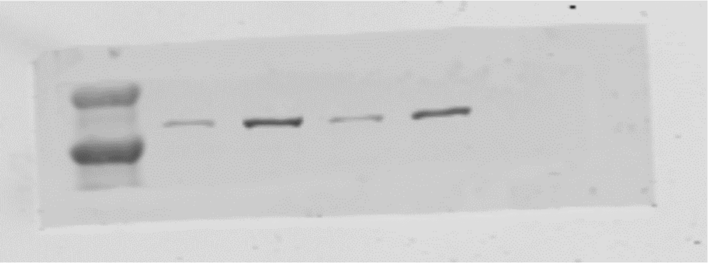

p-VASP C4-2

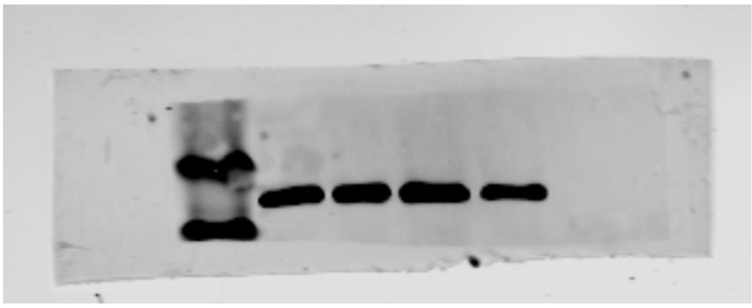

Caspase-3 DU145

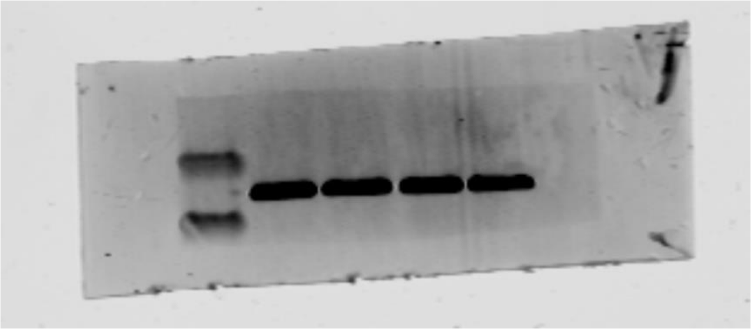

Caspase-3 C4-2

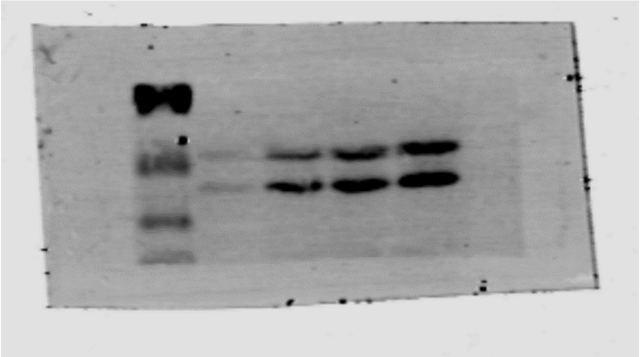

Cleaved Caspase-3 DU145

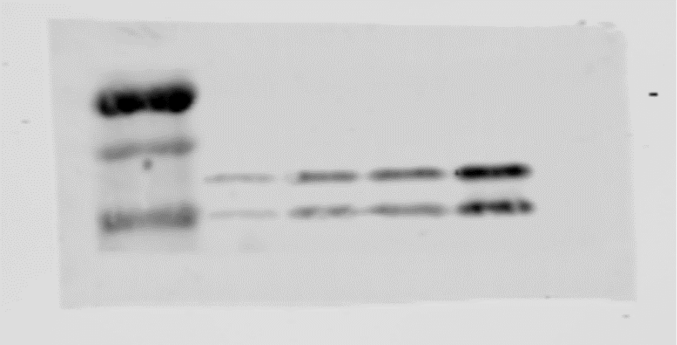

Cleaved Caspase-3 C4-2

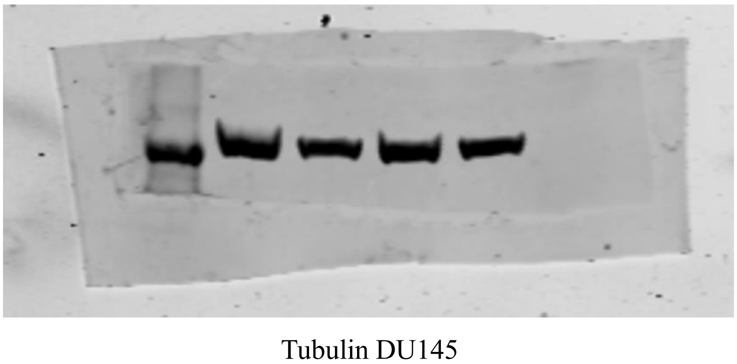

Tubulin DU145

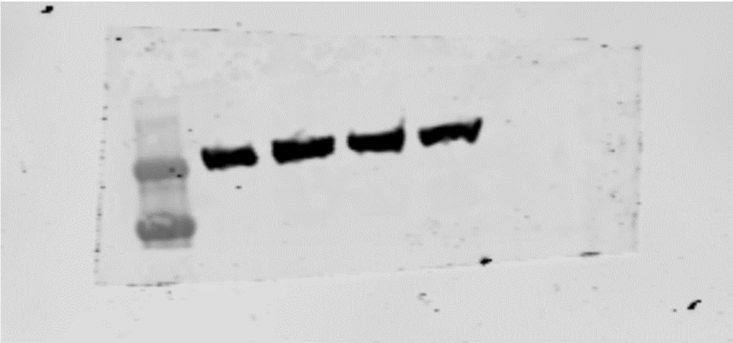

Tubulin C4-2

Supplementary Figure 5

h

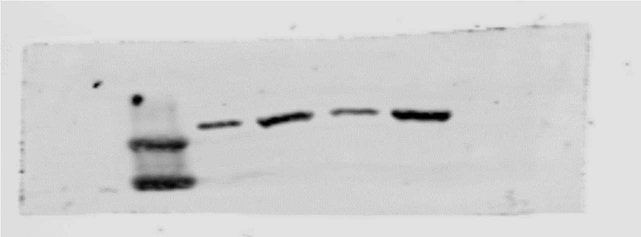

E2F1

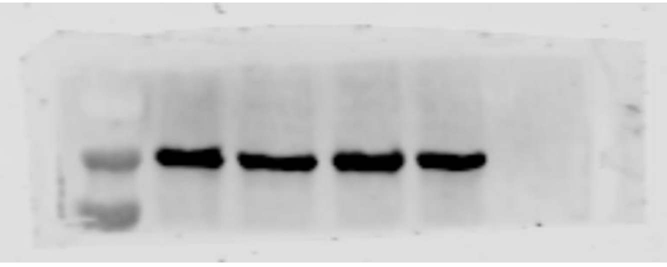

Tubulin

k

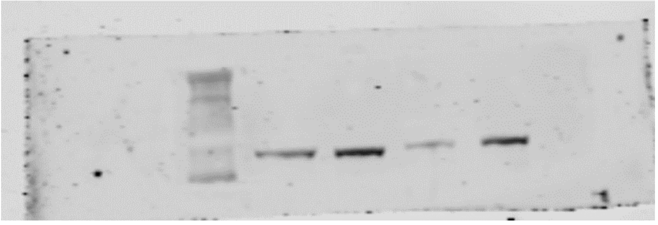

PROSER3

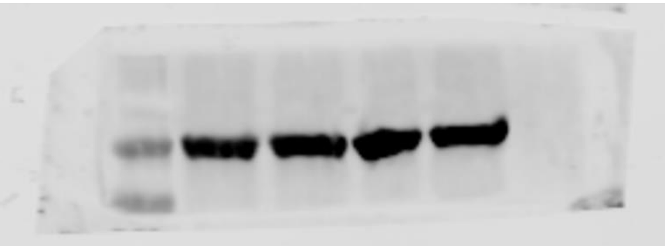

Tubulin

Supplementary Figure 6

b

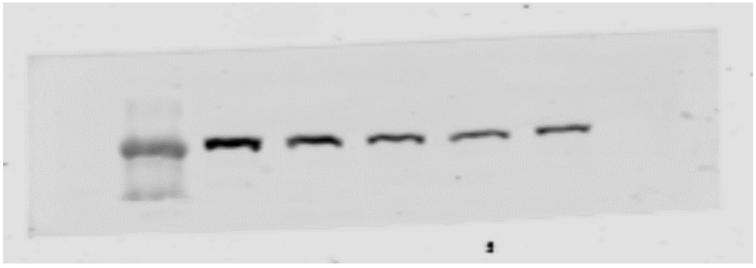

E2F1

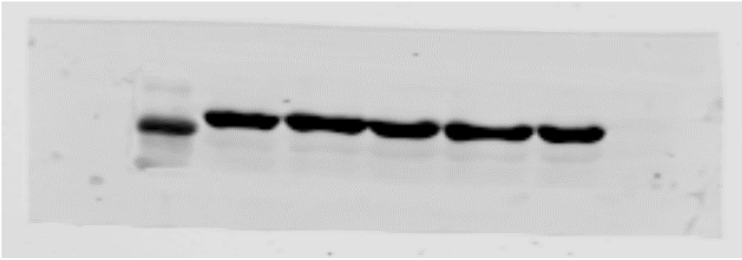

Tubulin

c

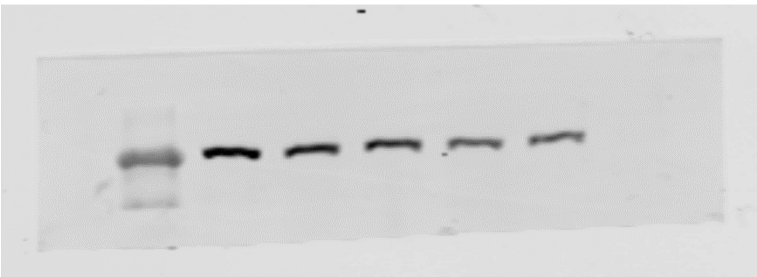

E2F1

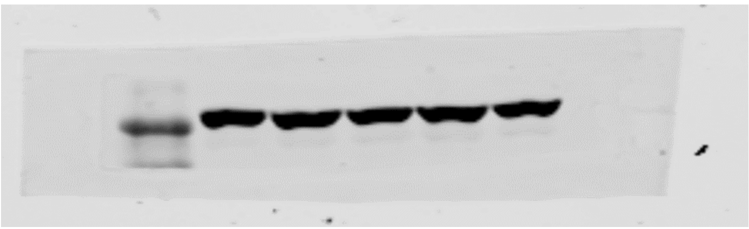

Tubulin

d

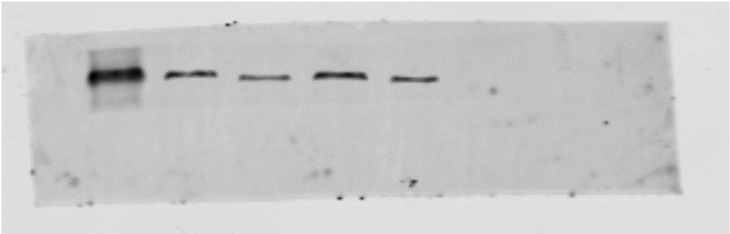

E2F1

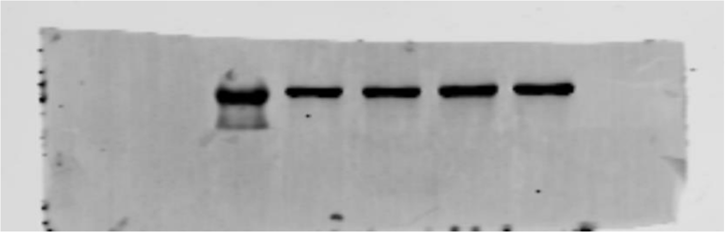

Tubulin

e

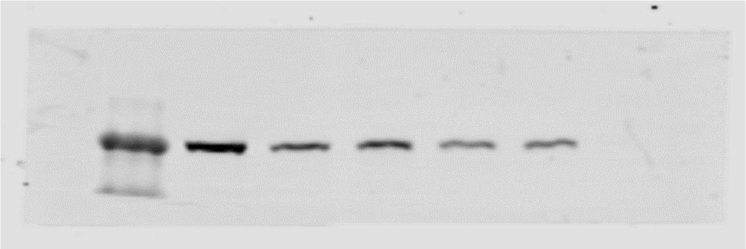

E2F1

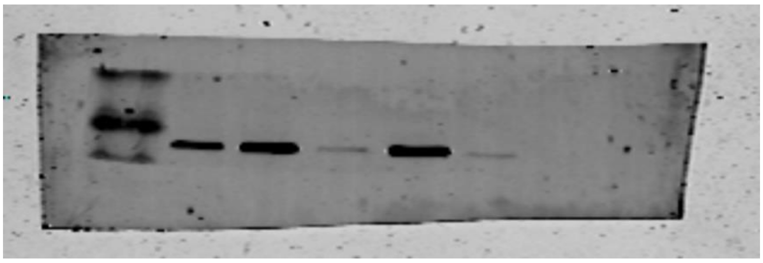

HSPB6

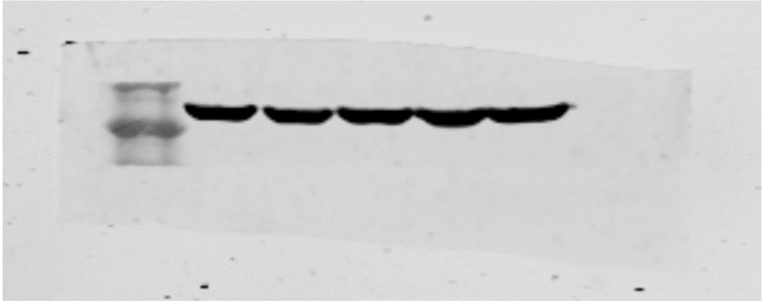

Caspase-3

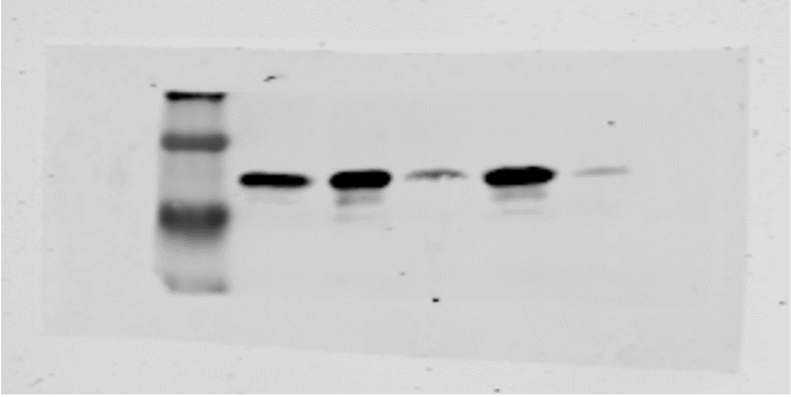

Cleaved Caspase-3

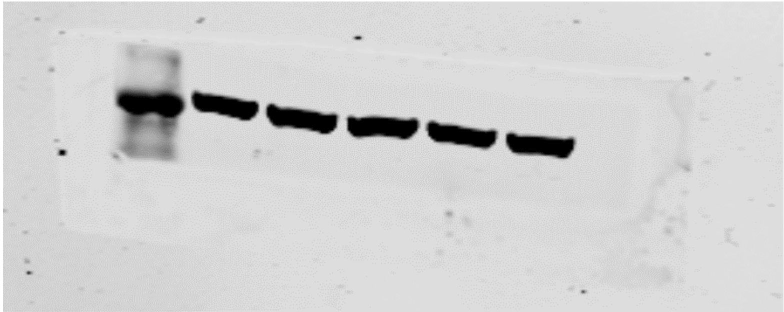

Tubulin

f

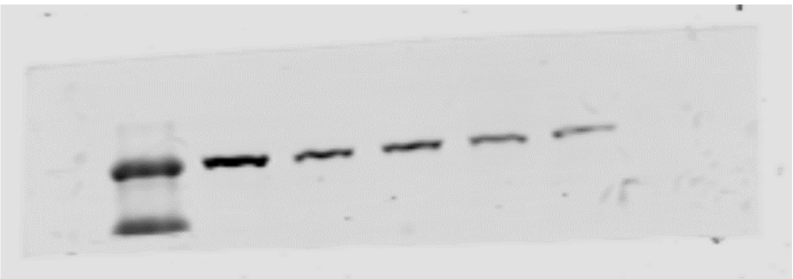

E2F1

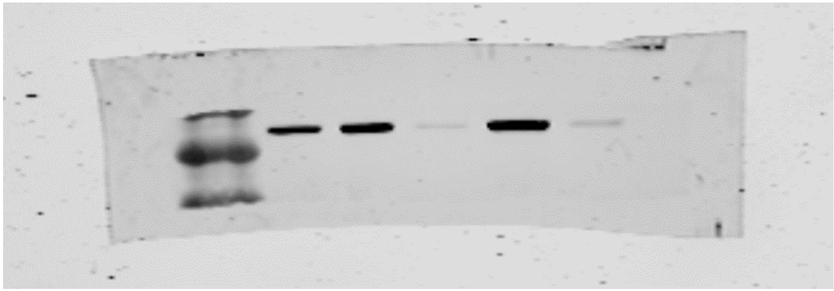

HSPB6

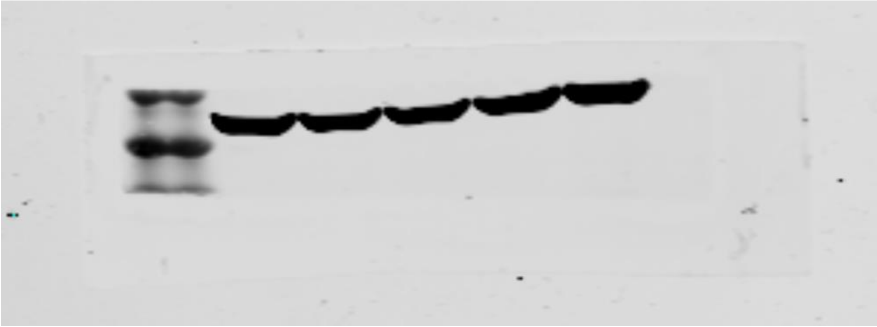

Caspase-3

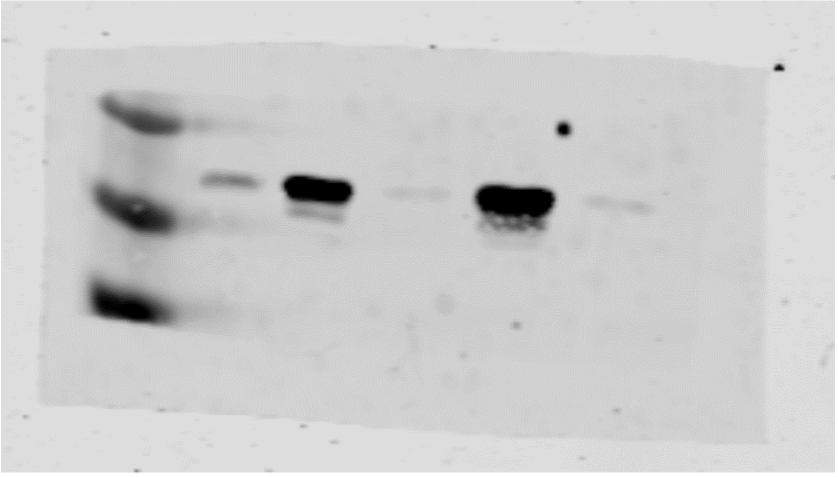

Cleaved Caspase-3

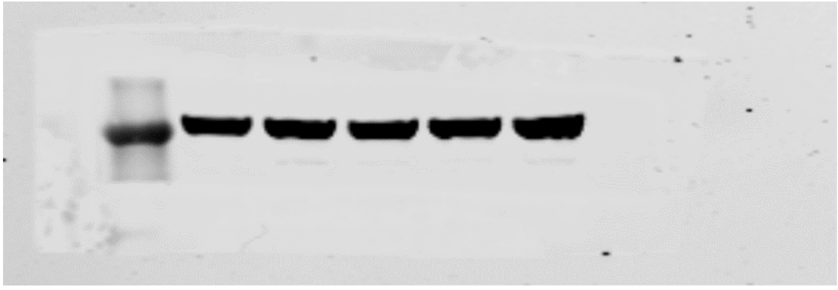

Tubulin
